# Inappropriate Neural Activity during a Sensitive Period in Embryogenesis Results in Persistent Seizure-like Behavior

**DOI:** 10.1016/j.cub.2015.09.040

**Published:** 2015-11-16

**Authors:** Carlo N.G. Giachello, Richard A. Baines

**Affiliations:** 1 Faculty of Life Sciences, University of Manchester, Manchester M13 9PT, UK

## Abstract

Maturation of neural circuits requires activity-dependent processes that underpin the emergence of appropriate behavior in the adult. It has been proposed that disruption of these events, during specific critical periods when they exert maximal influence, may lead to neurodevelopmental diseases, including epilepsy [1, 2, 3]. However, complexity of neurocircuitry, coupled with the lack of information on network formation in mammals, makes it difficult to directly investigate this hypothesis. Alternative models, including the fruit fly *Drosophila melanogaster*, show remarkable similarities between experimental seizure-like activity and clinical phenotypes [4, 5, 6]. In particular, a group of flies, termed bang-sensitive (bs) mutants have been extensively used to investigate the pathophysiological mechanisms underlying seizure [7, 8, 9, 10, 11, 12]. Seizure phenotype can be measured in larval stages using an electroshock assay, and this behavior in bs mutants is dramatically reduced following ingestion of typical anti-epileptic drugs (AEDs; [13]). In this study we describe a critical period of embryonic development in *Drosophila* during which manipulation of neural activity is sufficient to significantly influence seizure behavior at postembryonic stages. We show that inhibition of elevated activity, characteristic of bs seizure models, during the critical period is sufficient to suppress seizure. By contrast, increasing neuronal excitation during the same period in wild-type (WT) is sufficient to permanently induce a seizure behavior. Further, we show that induction of seizure in WT correlates with functional alteration of motoneuron inputs that is a characteristic of bs mutants. Induction of seizure is rescued by prior administration of AEDs, opening a new perspective for early drug intervention in the treatment of genetic epilepsy.

## Highlights

• Activity manipulation defines a critical period for circuit functionality
• Abnormal activity during the critical period induces seizure
• Early drug intervention prevents seizure occurrence at postembryonic stages
• Seizure behavior correlates with aberrant synaptic excitation of motoneurons

## Teaser

Giachello and Baines show that preventing abnormal neuronal activity during a critical period of *Drosophila* embryogenesis in genetic and chemical models of seizure is sufficient to prevent the emergence of seizure behavior.

## Results

### Inhibiting Neuronal Activity during Embryogenesis Prevents the Development of a Seizure Phenotype

We have previously shown that the presence of anti-epileptic drugs (AEDs), administered to developing embryos by feeding gravid females, is sufficient to prevent the emergence of seizure behavior in response to electroshock in mature bang-sensitive (bs) larvae (when AEDs are no longer detectable) [13]. We postulated that increased levels of excitatory synaptic activity observed in the CNS of bs mutant embryos are prevented in the presence of AEDs. Manipulation of early neural activity may, therefore, represent a route to control epileptogenesis. In support of this hypothesis, we find a direct correlation between temperature during embryogenesis and subsequent seizure duration in the *bang-senseless* (*bss*; [8, 14]) mutant (Figure S1). This suggests that temperature-sensitive events, for example, neuronal metabolism and/or circuit activity, are crucial during embryogenesis in determining seizure behavior.

To specifically investigate the contribution of neuronal activity, we used optogenetic tools halorhodopsin (eNpHR) and channelrhodopsin (ChR) to selectively modulate neuronal activity during embryogenesis. Whole-cell patch recordings from first instar larvae (L1) confirmed that ChR can depolarize *Drosophila* motoneurons to fire action potentials (APs; Figure S2A), as previously described [15]. Conversely, we found that the effect of eNpHR is stimulation time dependent (Figure S2B; see Supplemental Experimental Procedures). Brief stimulation (λ565 nm, 100 ms/1 Hz) induced a post-inhibitory rebound with significant AP firing (thus could be considered excitatory). Prolonged stimulation (λ565 nm, 600 ms/1 Hz) produced only inhibition of neuronal activity (considered to be inhibitory).

**Figure 1:**
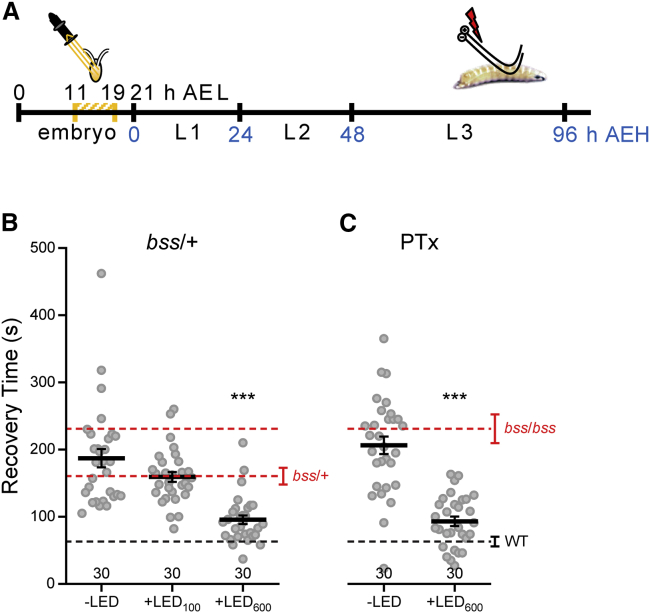
Preventing Neuronal Hyperexcitability during Embryogenesis Is Sufficient to Suppress Seizure (A) Schematic representation of the experimental procedure. Embryos expressed eNpHR in cholinergic neurons and were exposed to light (λ565 nm, 100 or 600 ms/1 Hz) between 11 and 19 hr AEL. Subsequent L3 larvae were tested for seizure behavior by electroshock. AEH, after embryo hatching. (B) Electroshock-induced seizure recovery time is significantly reduced in a genetic (*bss*) seizure mutant (*bss*/+; B19-GAL4/eNpHR) following inhibition of neural activity (600-ms light pulses) during embryogenesis (compare +LED_600_ to −LED). By contrast, short duration light pulses of 100 ms (+LED_100_), which are excitatory (see Figure S2), are without effect. The *bss* mutation is semi-dominant, conferring a weaker but still significant seizure phenotype in heterozygous progeny (*bss*/+). (C) Inhibition of neural activity in embryos exposed to the proconvulsant PTx is sufficient to significantly prevent seizure (compare +LED_600_ to −LED). Data are represented as mean ± SEM. ^∗∗∗^p < 0.001, Bonferroni’s post hoc test. Red and black dotted lines represent reference RTs (mean ± SEM) obtained from *bss*, *bss/+* and WT, respectively.

To manipulate neuronal activity in *bss* embryos, we expressed eNpHR in cholinergic neurons, which provide the primary excitatory synaptic drive in insect CNS. Embryos were collected and exposed to light between 11 and 19 hr after egg laying (AEL), and the resulting third instar larvae (L3) were electroshocked 4 days later (Figure 1A). We found that increasing neuronal inhibition with prolonged stimulation (λ565 nm, 600 ms/1 Hz) produced an almost total rescue of seizure behavior (+LED_600_, Figure 1B), with recovery times (RTs) significantly reduced to values typical of wild-type (WT). Conversely, shorter light pulses that elicit excitation (λ565 nm, 100 ms/1 Hz) had no effect on seizure phenotype (+LED_100_, Figure 1B). We repeated eNpHR-mediated manipulation in a drug-induced seizure model. Picrotoxin (PTx), a known proconvulsant, produces seizures when administered to *Drosophila* larvae [16, 17], or adults [18]. PTx exposure in WT embryos is sufficient to induce a seizure phenotype, mirroring the effect of the *bss* mutation (−LED, Figure 1C). This effect of PTx was prevented by activation of eNpHR (λ565 nm, 600 ms/1 Hz) during the embryonic critical period (+LED_600_, Figure 1C). Our manipulations of neural activity during embryogenesis were limited to effects in subsequent larvae (Table S1). Adult flies derived from manipulated embryos show no obvious differences to their non-manipulated counterparts. This is expected and, indeed, is an important control. A lack of carryover to the adult is almost certainly because during metamorphosis (i.e., larva to adult transition), the larval CNS undergoes profound remodeling, involving a significant second wave of neurogenesis and de novo neural circuit formation.

### Manipulation of Neuronal Activity during a Critical Period in WT Is Sufficient to Confer a Seizure Phenotype at Postembryonic Stages

**Figure 2:**
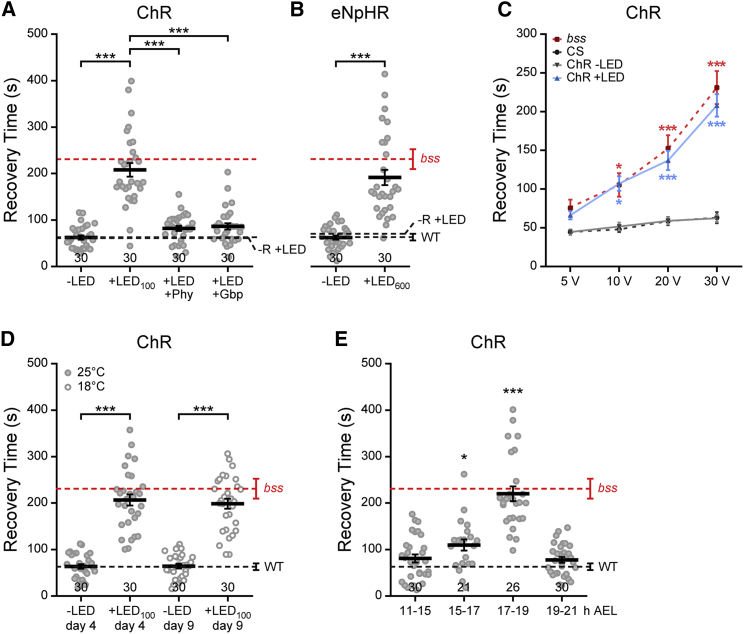
A Critical Period to Influence Seizure (A and B) RTs measured from L3 that pan-neuronally expressed either ChR (λ470 nm, 100 ms/1 Hz) or eNpHR (λ565 nm, 600 ms/1 Hz) and exposed to light during 11–19 hr AEL of embryogenesis. In both manipulations, disturbing neural activity (+LED) is sufficient to increase the RT of L3 in response to electroshock. Controls were not exposed to light (−LED). The presence of AEDs, Phy (0.4 mg/ml) and Gbp (0.1 mg/ml), during embryogenesis prevents the induction of a seizure phenotype. In order to exclude an unspecific effect of the LED stimulation, embryos were optically manipulated in absence of *all-trans*-retinal (−R+LED, black dotted lines). The RT (mean ± SEM) for homozygous *bss* is shown for reference (red dotted lines). (C) Seizure threshold is lower in L3 derived from manipulated embryos. Seizure response to varying voltages shows a lower threshold for *bss* [8, 19]. Similarly, larvae in which activity was manipulated during embryogenesis (+LED) require a lower voltage to exhibit a significant increase in RT compared to controls (−LED and WT, n = 20 in each group). (D) The effect of ChR activation is independent of developmental time. To extend duration of larval stage, larvae were maintained at either 25°C (4 days) or 18°C (9 days) until L3. Two-way ANOVA shows a significant effect of LED treatment (F_(1,116)_ = 249.39, p < 0.001), but no effect for developmental time (F_(1,116)_ = 0.18, p = 0.67). (E) Temporally controlled experiments indicate that manipulation of neuronal activity (ChR, λ470 nm, 100 ms/1 Hz) between 17 and 19 hr AEL is sufficient to induce maximal seizure duration at L3. Data (A–E) are represented as mean ± SEM. ^∗^p < 0.05 and ^∗∗∗^p < 0.001, Bonferroni’s post hoc test. The number of tested larvae is shown above the x axis. Red and black dotted lines represent reference RTs (mean ± SEM) obtained from homozygous *bss* and WT, respectively.

The rescue of seizure in L3 that we observed by eNpHR-driven inhibition during embryonic neurogenesis is indicative that neural circuit function can be altered by abnormal levels of synaptic excitation that occur during embryogenesis. A powerful test of this is to increase neural activity in WT embryos and assess for increased seizure behavior at L3. Thus, we expressed ChR pan-neuronally in WT embryos and exposed them to blue light (λ470 nm, 100 ms/1 Hz) between 11 and 19 hr AEL. L3 derived from treated embryos exhibited a marked increase in RT to electroshock (Figure 2A), reaching values comparable with those observed in *bss*. Lower frequency stimulation (0.1 Hz) produced seizures of reduced duration, and stimulation at 0.01 Hz was ineffective (see Supplemental Experimental Procedures). The effect to seizure is, moreover, prevented by feeding the AEDs phenytoin (Phy) or gabapentin (Gbp) to gravid adult females that produced the embryos (Figure 2A). We also tested the effect of increased neuronal inhibition during embryogenesis by expression and activation of eNpHR (λ565 nm, 600 ms/1 Hz) in WT embryos. Surprisingly, subsequent L3 showed an identical heightened RT to electroshock (Figure 2B). Seizure threshold was assessed by applying electroshock stimuli at increasing voltages (from 5 to 30 V, Figure 2C). The *bss* strain exhibits a clear reduction in threshold [8, 19]. A similar reduced threshold was observed in WT L3 optogenetically treated during embryogenesis, suggesting an increase in seizure susceptibility. Taken together, these data suggest that disturbance of normal activity patterns during embryogenesis, rather than just increased excitation, is sufficient to produce a reduced threshold and increased duration of seizure in postembryonic larvae.

In order to test how permanent the alteration to circuit function is, we took advantage of the fact that larval development of *Drosophila* is temperature dependent [20]. Newly hatched L1 were collected after optical manipulation during embryogenesis at 25°C and maintained at either 25°C (4 days) or 18°C (9 days) until the L3 wandering stage. We measured comparable RT values in L3 at both 4 and 9 days, respectively, indicating that this induced seizure behavior is independent of development time and persists through the larval stage (Figure 2D). Optogenetics also facilitates the determination of critical periods. To define a critical period of sensitivity for the effect of altered neural activity with regard to seizure, we performed temporally controlled experiments. We found that perturbing neural activity between 17 and 19 hr AEL is optimal to destabilize CNS function, resulting in a significant increase in RT when tested at L3 (Figure 2E). Again, this effect was limited to L3 and did not carry over to the adult stage (Table S1). Identical results were observed with eNpHR (data not shown).

A significant goal toward a better understanding of epileptogenesis is the determination of whether abnormal activity of neuron number or neuron type is important. To address this, we utilized a range of neuron-specific GAL4 drivers to express eNpHR in the embryonic CNS. Short light pulses (λ565 nm, 100 ms/1 Hz, Figure S2B) were applied in order to trigger excitatory firing to a similar extent to ChR (exposure to a yellow instead of blue light does not require the use of a *cry* null background; see Supplemental Experimental Procedures). Although the response of different cell types to optogenetic manipulation is problematic to accurately predict, our results implicate a major involvement of both sensory and interneuron components of the larval CNS rather than a contribution by motoneurons (for details, see Table S2). Using a subtractive approach (ElaV^C155^-GAL4; tsh-GAL80), we tested the relative contribution of all neurons in the brain lobes compared to those in the ventral nerve cord (VNC). Manipulation of the former was not able to produce a bs phenotype when tested in WT L3, indicative that seizures arise following manipulation of the locomotor circuits located in the VNC. Finally, no effects were observed when expression was limited to glia (Table S2). We conclude that efficacy of effect is not overtly dependent on the number of manipulated neurons but rather on their specific connectivity and/or function.

### Seizure Phenotype Correlates with Aberrant Synaptic Excitation

**Figure 3:**
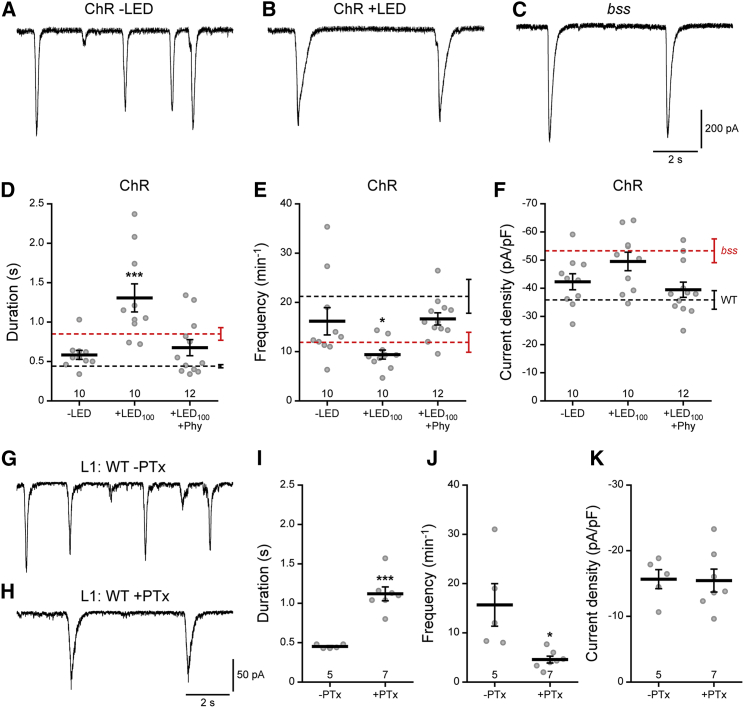
Manipulation of Neuronal Activity Results in an Increase in Synaptic Excitation (A–C) Whole-cell patch recordings of SRCs from identified L3 aCC/RP2 motoneurons. L3 were derived from WT embryos in which ChR was expressed pan-neuronally (B, ChR+LED) and activated between 11 and 19 hr AEL (λ470 nm, 100 ms/1 Hz). Controls shown were not exposed to light (A, ChR-LED) or are from *bss* L3 (C). (D and E) Optogenetic manipulation of embryos in which ChR was activated (+LED) shows SRCs at L3 that are increased in duration and reduced in frequency to mirror values recorded in homozygous *bss*. Exposure to Phy during embryogenesis is sufficient to block change to SRC duration and frequency, showing values comparable to those obtained from WT. (F) SRC amplitudes, normalized to cell capacitance, are not statistically different. (G and H) Whole-cell patch recordings of SRCs from identified L1 aCC/RP2 motoneurons. L1 were derived from WT embryos exposed to PTx by feeding gravid adult females. (I and J) PTx exposure during embryogenesis produces a statistically significant increase in synaptic input duration and a decrease in input frequency, as described for *bss*. (K) SRC amplitudes, normalized to cell capacitance, are not statistically different. Data (D–K) are represented as mean ± SEM. ^∗^p < 0.05 and ^∗∗∗^p < 0.001, Bonferroni’s post hoc test. The number of tested larvae is indicated above the x axis. Red and black dotted lines (D–F) represent reference RTs (mean ± SEM) obtained in *bss* and WT, respectively.

A plausible mechanism for prolonged duration of seizure recovery is an increased, and/or uncoordinated, synaptic excitation of larval motoneurons. The dorsal motoneurons, aCC and RP2, receive identical cholinergic excitatory synaptic inputs [21] termed spontaneous rhythmic currents (SRCs; Figure 3) [22]. Compared to WT, SRCs recorded in the bs mutants *slamdance* (*sda*; [13]) and *bss* (Figure 3C) were increased in both amplitude and, in particular, duration. By contrast SRC frequency is dramatically reduced. SRCs recorded from aCC/RP2 in L3 derived from manipulated embryos pan-neuronally expressing ChR (ElaV^C155^-GAL4) similarly exhibited SRCs with longer duration (Figure 3D) and decreased frequency (Figure 3E). SRC amplitudes, while larger, were not statistically different (Figure 3F). Changes to both duration and frequency were completely reversed by early administration of Phy prior to optogenetic manipulation, by feeding drug to gravid females. Electrophysiological recordings from newly hatched WT L1 derived from adult females fed PTx showed a similar increase in SRC duration and a decrease in frequency with no alteration in amplitude (Figures 3G–3K). Thus, the changes to network activity induced by optogenetic manipulation of the CNS of developing WT embryos results in modifications to neuronal and network properties that are characteristic of bs mutants, or PTx-exposure. Early drug intervention shows that this alteration must occur at a defined period for a heightened seizure phenotype to occur postembryonically. However, it should be noted that we record from only two motoneurons, and how synaptic excitation of the other motoneurons in the larval CNS is affected remains to be determined.

The observed changes to SRC kinetics may be diagnostic of an inability to confine activity levels within the locomotor circuit, consistent with an inability of activity-dependent homeostasis to fully constrain activity levels in this circuit. To determine whether homeostasis is operative under these conditions, we analyzed firing responses of aCC/RP2 in response to injection of depolarizing current. These revealed reduced membrane excitability (Figure S3), similar to the reduction previously reported in *sda* [13]. Thus, we conclude that while homeostasis, at least in motoneurons, is active, it cannot fully compensate for the change to network excitation that occurs due to activity manipulation during the embryonic critical period.

## Discussion

Currently available treatments for seizure are inadequate. AEDs alleviate seizure occurrence and severity but offer no cure. Recently, optogenetic tools have been successfully employed in rodents to inhibit epileptiform activity both in slice [23, 24] and in vivo [25, 26]. Promising results have been achieved by combining eNpHR with on-line seizure detection in order to reduce seizure in an on-demand fashion [27, 28, 29, 30]. However, these technologies are still antiepileptic and do not modify the underlying disease-causing mechanisms (i.e., antiepileptogenic). Here, we present evidence that a time-controlled intervention may prevent the onset of seizure, defining a critical period where disturbance of neuronal activity manifests in a heightened response to electroshock. This period coincides precisely with the time window where locomotor circuits are first functional. APs in motoneurons and coordinated body-wall muscle movements first appear at 17 hr AEL [31]. This period has also been determined sensitive for maturation of coordinated motor function in *Drosophila* embryos [32]. Nonetheless, a big challenge still remains to translate this discovery into models evolutionarily closer to humans. In placental mammals, less is known about the time course of neurogenesis occurring during prenatal development. We speculate that in newly formed neuronal networks, there is a time window during which neurons integrate into circuitry and use endogenous activity to refine their properties (i.e., intrinsic excitability, synaptic strength, balance between excitatory and inhibitory synapses, etc.).

It is well established that *Drosophila* motoneurons, like their mammalian counterparts, are able to homeostatically adjust intrinsic excitability to compensate for changing levels of synaptic excitation, in this case from cholinergic presynaptic interneurons [21, 33]. Our results are consistent with a failure of the activity-dependent processes required to incorporate homeostatic limits, based on the dynamic range of activity to which neurons are exposed, during that period. Further investigation of this period may offer the exciting possibility to uncover key molecular components required to define a homeostatic set point. Once set, these limits are seemingly permanent and likely provide the upper and lower extremes for ongoing homeostatic mechanisms that operate in the mature CNS. Capping of activity, with AEDs or optogenetics, during the critical period is sufficient to prevent the emergence of a seizure phenotype in characterized seizure models. If conserved across species, the presence of a critical period in the later stages of neurogenesis could be exploited for therapeutic purposes in humans.

## Author Contributions

C.N.G.G. and R.A.B. designed experiments. C.N.G.G. performed experiments and analyzed data. C.N.G.G. and R.A.B. wrote the paper.

## Supplemental Information

Document S1. Supplemental Experimental Procedures, Figures S1–S3, and Tables S1 and S2

Document S2. Article plus Supplemental Information
