# Supplementary material for: Inappropriate Neural Activity during a Sensitive Period in Embryogenesis Results in Persistent Seizure-like Behavior
Source: Curr Biol. 2015 Nov 16;25(22):2964–8. doi: 10.1016/j.cub.2015.09.040 (PMC4651905; doi:10.1016/j.cub.2015.09.040)
Supplement: Document S1. Supplemental Experimental Procedures, Figures S1–S3, and Tables S1 and S2 [file mmc1.pdf]

Current Biology
Supplemental Information

Inappropriate Neural Activity during a Sensitive Period in Embryogenesis Results in Persistent Seizure-like Behavior

Carlo N.G. Giachello and Richard A. Baines

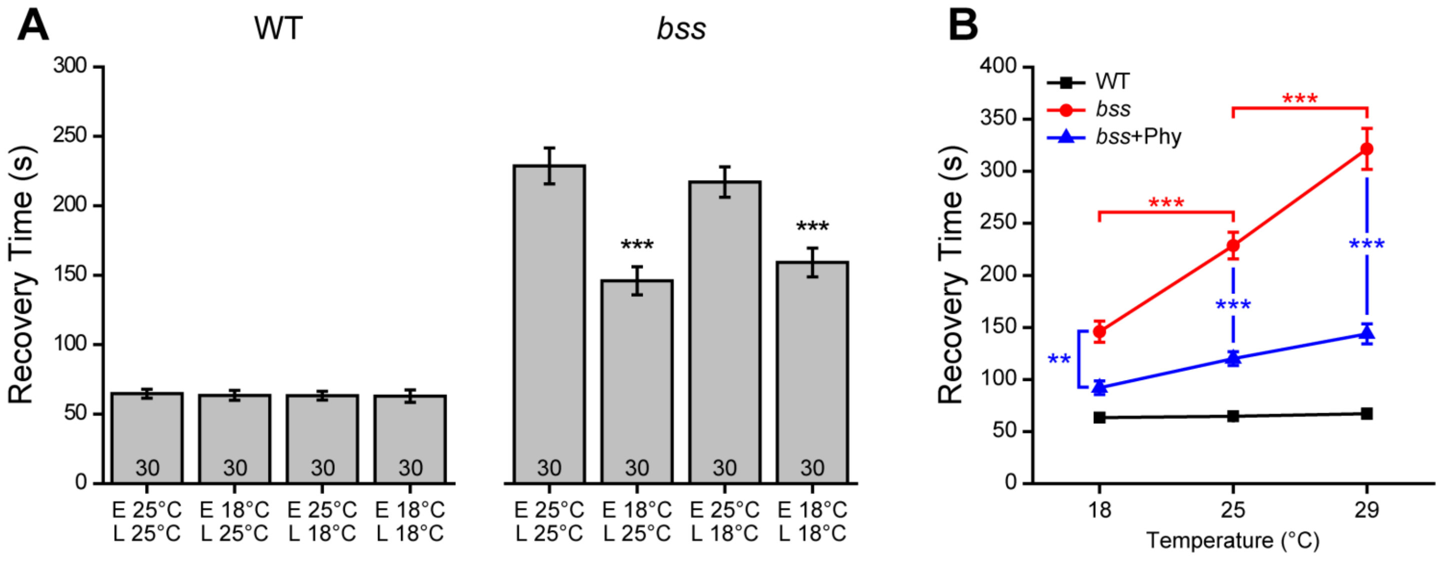

Figure S1. Developmental temperature during embryogenesis affects seizure severity

(**A**) RTs measured at L3 from WT and homozygous *bss* pre-incubated at different temperatures during both embryonic (E) and larval (L) stages. Only *bss* L3 derived from embryos maintained at 18°C, compared to either normal (25°C) or elevated (29°C) temperature, showed a statistically significant recovery of seizure-behavior. By contrast, altering temperature after hatching had no effect on seizure severity. The number of tested larvae is indicated in the bars. (**B**) RTs from WT and homozygous *bss* incubated at different temperatures (18°C, 25°C and 29°C) during embryogenesis only. Newly-hatched L1 from all groups were maintained at 25°C until L3. Only *bss* embryos maintained at 18°C show significantly reduced RT ($F_{(2, 261)} = 223.70$, $p < 0.001$, n = 30 in each group), suggesting that temperature-sensitive events, such as neuronal metabolism and/or circuit activity (see Figure 1B), are implicated in determining seizure-behavior. *bss* larvae treated with phenytoin (Phy) during embryogenesis exhibited a reduced dependency on temperature and RTs were lower with respect to the untreated *bss* line, indicating a beneficial treatment of the drug. To determine AED sensitivity, *bss* embryos were exposed to Phy by feeding gravid adult females. Data (A and B) are represented as mean ± SEM. *** = p < 0.001 with respect to the E25°C-L25°C group, Bonferroni's *post-hoc* test.

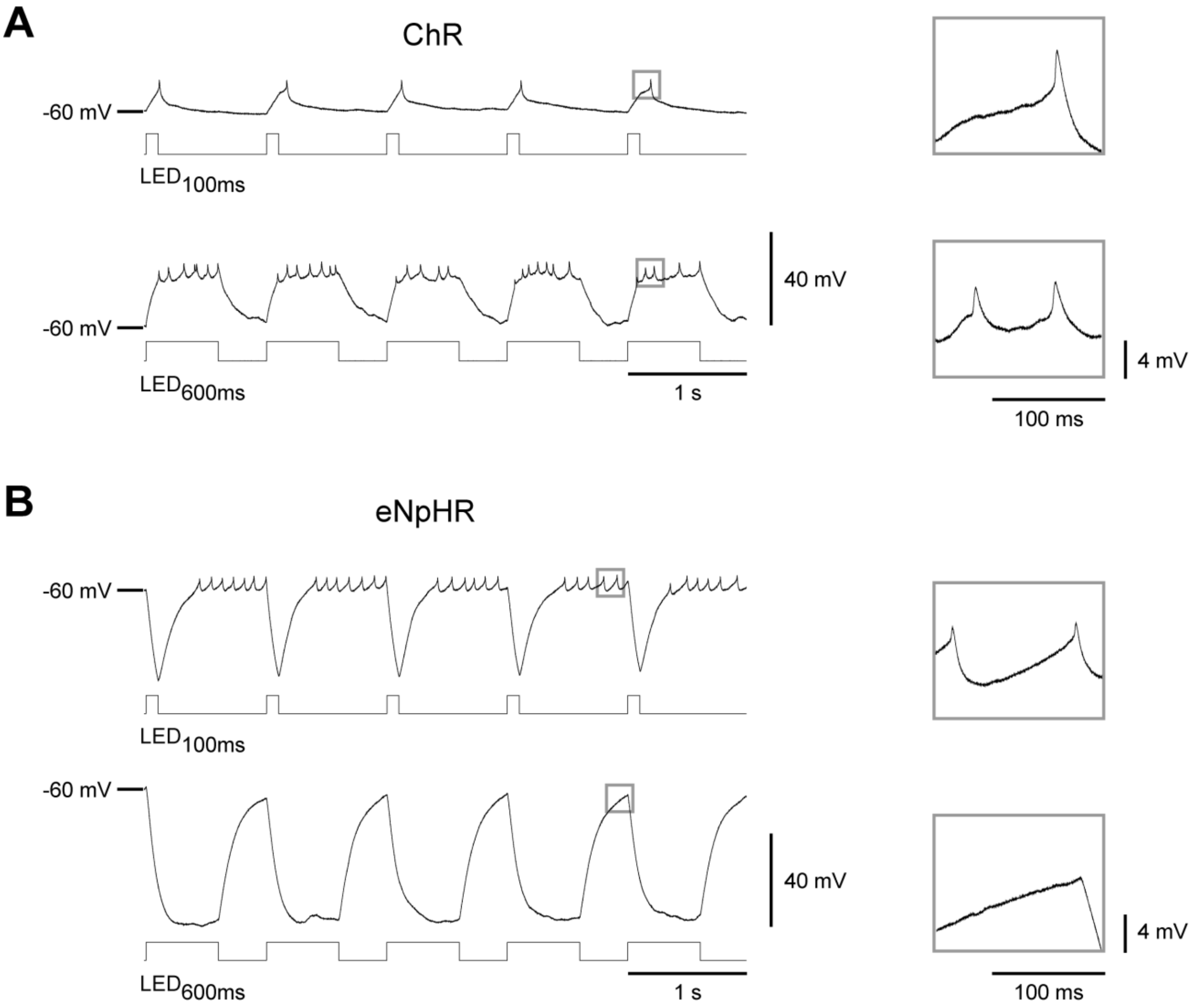

Figure S2. The effect of eNpHR is stimulation-time dependent

(**A**) Whole-cell patch recordings from aCC/RP2 motoneurons in newly-hatched L1 (~21 hours AEL) showing membrane potential changes due to activation of ChR (λ470 nm, 100 or 600 ms/1 Hz). Single (1.37 ± 0.26 APs per light pulse, n = 5) or multiple (4.97 ± 0.56 APs per light pulse, n = 5) action potentials (APs) are elicited following ChR activation for either short (100 ms) or long (600 ms) periods, respectively. (**B**) In eNpHR-expressing neurons short duration (λ565 nm, 100 ms/1 Hz) activation produced brief hyperpolarization events that gave rise to rebound AP firing (4.42 ± 1.34 APs per light pulse, n = 8), due to anodal-break excitation. By contrast, prolonged photo-activation (λ565 nm, 600 ms/1 Hz) produced only inhibitory hyperpolarizing changes to the membrane potential with no rebound firing (0.36 ± 0.31 APs per light pulse, n = 8). Indeed, pulse duration (100 *vs*. 600 ms) affects the ability of eNpHR to suppress seizure in our model (see Figure 1B). Inset shows magnified images of the light-evoked APs.

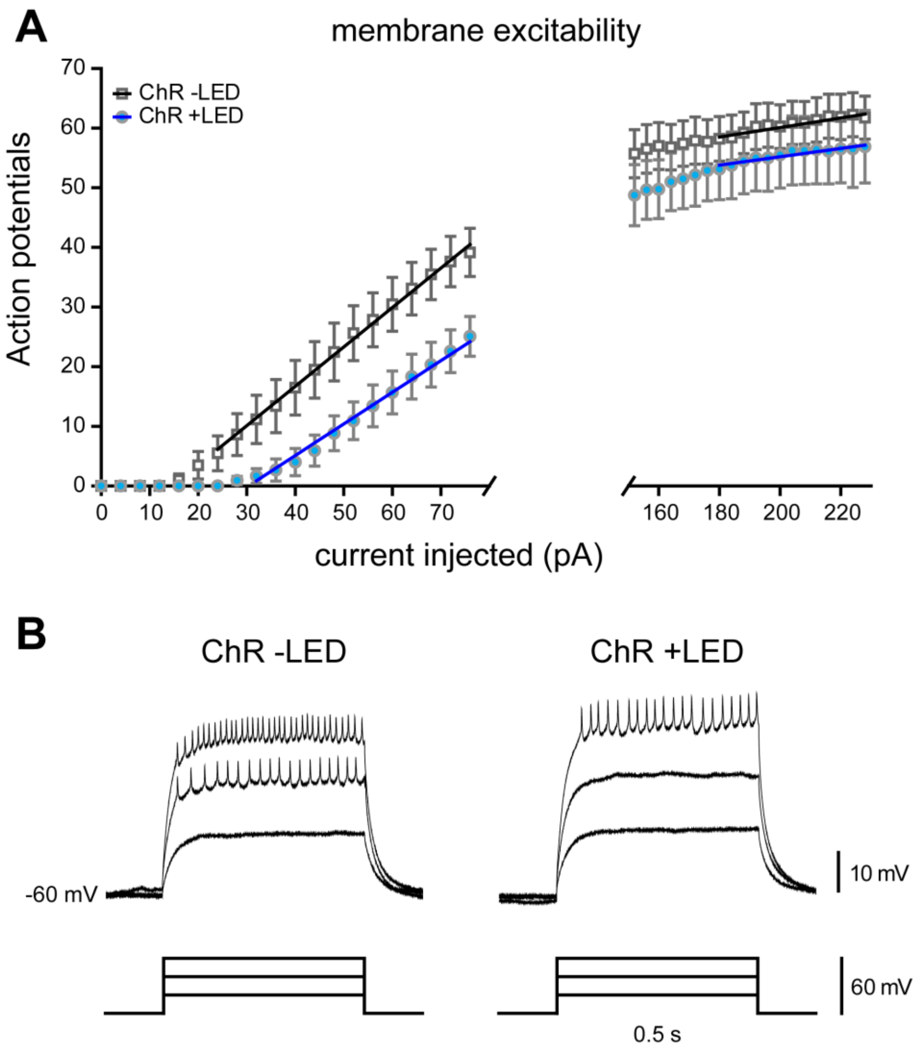

Figure S3. L3 motoneurons derived from optogenetically-manipulated embryos exhibit reduced membrane excitability

(**A**) Changes in membrane excitability of L3 aCC/RP2 motoneurons, following the increased synaptic excitation (see Figure 3), were determined by injection of constant current (4 pA steps/0.5 s). The current-firing relationship was evaluated by linear regression. The linear part (28-76 pA) and the plateau (180-228 pA) of the curve were analyzed separately. The number of APs fired is significantly less in manipulated (+LED, n = 12) compared to control (-LED, n = 13) L3. The fitted lines exhibit similar slope values (linear part: 0.53 ± 0.02 *vs*. 0.66 ± 0.01, +LED *vs*. -LED, respectively; and plateau: 0.07 ± 0.01 *vs*. 0.08 ± 0.01, +LED *vs*. -LED, respectively), but different intercepts considering both the rising (-16.10 ± 0.97 *vs*. -9.69 ± 0.65, +LED *vs*. -LED, respectively, $p < 0.001$) and plateau (41.19 ± 1.62 *vs*. 44.10 ± 1.68, +LED *vs*. -LED, respectively, $p = 0.004$) elements of the curves. No difference in resting membrane potential, input resistance or capacitance was observed (data not shown). Data are represented as mean ± SEM. (**B**) Representative traces showing firing of APs by successively greater depolarizing current injections (20, 40, and 60 pA/0.5 s) in aCC.

Table S1. Neuronal remodeling during pupation resets the effect of optogenetic manipulation.

	Treatment	n	Mean ± SEM	p-value
bss/+	eNpHR -LED	60	77.67 ± 14.89 s	
bss/+	eNpHR +LED_{600ms}	60	67.83 ± 9.43 s	n.s.
bss/+	eNpHR +LED_{100ms}	60	73.50 ± 13.34 s	n.s.
WT + PTx	eNpHR -LED	100	0.73 ± 0.05 s	
WT + PTx	eNpHR +LED_{600ms}	100	0.71 ± 0.06 s	n.s.
WT	ChR -$LED_{11\text{-}15\,h}$	15	0.73 ± 0.14 s	
WT	ChR +$LED_{11\text{-}15\,h}$	18	0.71 ± 0.07 s	n.s.
WT	ChR -$LED_{15\text{-}17\,h}$	41	0.75 ± 0.15 s	
WT	ChR +$LED_{15\text{-}17\,h}$	47	0.73 ± 0.11 s	n.s.
WT	ChR -$LED_{17\text{-}19\,h}$	49	0.77 ± 0.11 s	
WT	ChR +$LED_{17\text{-}19\,h}$	54	0.75 ± 0.07 s	n.s.
WT	ChR -$LED_{19\text{-}21\,h}$	64	0.68 ± 0.11 s	
WT	ChR +$LED_{19\text{-}21\,h}$	41	0.70 ± 0.10 s	n.s.
Reference values				
WT		80	0.70 ± 0.10 s	
bss/bss		100	242.66 ± 25.30 s	
bss/+		90	88.39 ± 28.61 s	

RTs measured by vortexing adult flies, derived from manipulated embryos. (**A**) The ability of eNpHR-activation to rescue seizure in L3 carrying the *bss* mutation, is not maintained in adults flies derived from these larvae (cf. Figure 1B). (**B**) The effect of embryonic exposure to PTx to induce seizure in L3, is also not carried forward to the adult (cf. Figure 1C). This loss of effect is not influenced by activation of eNpHR during embryogenesis. (**C**) Similarly, neuronal remodeling during pupation completely rescues the ChR-induced seizure-phenotype observed in L3 (cf. Figure 2E).

Table S2. Different neuronal subpopulations contribute unequally to seizure predisposition.

GAL4 lines (neuron types)	Treatment	n	Mean ± SEM	p-value
$ElaV^{C155}$-GAL4 (all neurons) [S1]	-LED	18	70.22 ± 8.72 s	
	$+LED_{100ms}$	25	177.08 ± 16.48 s	<0.001
B19-GAL4 (cholinergic) [S2]	-LED	30	62.73 ± 3.86 s	
	$+LED_{100ms}$	30	187.57 ± 16.86 s	<0.001
vGat-GAL4 (GABAergic) [S3]	-LED	30	60.50 ± 5.19 s	
	$+LED_{100ms}$	30	163.03 ± 12.78 s	<0.001
TH-GAL4 (dopaminergic)[S4]	-LED	30	63.17 ± 3.95 s	
	$+LED_{100ms}$	30	108.93 ± 10.95 s	<0.001
TRH-GAL4 (serotonergic) [S5]	-LED	30	60.37 ± 4.27 s	
	$+LED_{100ms}$	30	100.97 ± 11.45 s	<0.001
OK371-GAL4 (glutamatergic) [S6]	-LED	30	59.87 ± 4.30 s	
	$+LED_{100ms}$	30	123.27 ± 7.73 s	<0.001
OK6-GAL4 (motoneurons) [S7]	-LED	30	62.27 ± 3.98 s	
	$+LED_{100ms}$	30	73.30 ± 4.42 s	n.s.
tdc2-GAL4 (tyraminergic/octopaminergic) [S8]	-LED	30	64.47 ± 3.72 s	
	$+LED_{100ms}$	30	74.17 ± 6.13 s	n.s.
386Y-GAL4 (peptidergic) [S9]	-LED	30	61.87 ± 4.40 s	
	$+LED_{100ms}$	30	93.30 ± 7.20 s	<0.001
Repo-GAL4 (glia cells) [S10]	-LED	30	62.73 ± 3.86 s	
	$+LED_{100ms}$	30	74.83 ± 5.82 s	n.s.
P0163-GAL4 (sensory) [S11]	-LED	30	60.13 ± 4.14 s	
	$+LED_{100ms}$	30	121.50 ± 12.67 s	<0.001
cry-GAL4 (Cry-positive) [S12]	-LED	30	63.83 ± 4.41 s	
	$+LED_{100ms}$	30	87.43 ± 6.87 s	0.005
$ElaV^{C155}$-GAL4;tsh-GAL80 (brain lobes)	-LED	30	60.67 ± 3.01 s	
	$+LED_{100ms}$	25	52.96 ± 6.64 s	n.s.

RTs of L3 to electroshock derived from embryos in which neuronal activity was manipulated by expression of eNpHR in different neuron types. Embryos were exposed to light (λ565 nm, 100 ms/1 Hz) between 11-19 hours AEL (see Figure 1A). In order to exclude an unspecific effect of the LED stimulation, embryos were also optically manipulated in absence of *all-trans*-retinal

(data not shown). Data were statistically compared to the corresponding -LED group using the Student's *t*-test.

Collectively, these data implicate the major neurotransmitter systems, e.g. cholinergic (B19), GABAergic (vGAT), glutamatergic (OK371), serotonergic (TRH), dopaminergic (TH) and peptidergic (386Y) to contribute to increase seizure duration. By contrast, increased activity in neurons directly involved in locomotion, i.e. motoneurons (OK6) and octopaminergic (tdc2) modulatory neurons was without effect. Motoneurons are glutamatergic and are also labeled by OK371 [S6]. By subtraction, we conclude that the effect of OK371-driven expression is achieved through manipulation of activity of the small population of glutamatergic interneurons (6 for each hemisegment) present in the embryonic CNS [S6]. We also observed increased seizure-like behavior following activity-manipulation of peripheral sensory inputs (P0163), which presumably contribute to levels of excitation centrally and influence the frequency of episodic activity in the developing motor network [S13]. Recent evidence indicates that the activation of Cry, a blue light-sensing photoreceptor, during embryogenesis, is sufficient to increase the seizure-phenotype of *Drosophila* WT L3 [S14]. Hence, we selectively manipulated the activity of Cry-positive neurons, obtaining a mild, but still significant, increase in RT. Just 6 Cry-positive neurons are present in the L3 CNS [S15, 16], indicating that activity-manipulation of even a small subset of neurons is sufficient to give a detectable effect. Finally, using a subtractive approach (ElaV^{C155}-GAL4; tsh-GAL80), we tested the relative contribution of all neurons in the brain lobes compared to those in the ventral nerve cord (VNC). Manipulation of the former was not able to produce a bs-phenotype when tested in WT L3 indicative that seizures arise following manipulation of the locomotor circuits located in the VNC. Finally, no effects were observed when expression was limited to glia (Repo).

SUPPLEMENTAL EXPERIMENTAL PROCEDURES

Fly stocks

Flies were maintained on standard corn meal medium at 25°C. Canton S flies from Bloomington *Drosophila* Stock Center was used as WT. Bang-sensitive flies used in this study were *bang senseless*[1] kindly provided by Kevin O'Dell. The following fly stocks were kindly provided by the respective researchers: channelrhodopsin w*; UAS-ChR2$_{H134R}$ (Stefan Pulver; [S17]), halorhodopsin UAS-eNpHR::YFP-50C; UAS-eNpHR::YFP-19C, UAS-eNpHR::YFP-34B (Akinao Nose; [S18]), B19-GAL4 (Paul M. Salvaterra), vGat-GAL4 (Justin Blau), TH-GAL4 and tdc2-GAL4 (Chris Elliott), OK6-GAL4 (Andreas Prokop), Repo-GAL4 (Angela Giangrande), P0163-GAL4 (Matthias Landgraf), cry-GAL4-19 (Ralf Stanewsky). All other lines were obtained from the Bloomington *Drosophila* Stock Center.

Previously published results indicate that embryonic exposure to blue light is sufficient to increase the RT in third instar larvae (L3) due to the activation of endogenous cryptochrome (Cry) [S14]. In this study, all the experiments involving blue light were performed in a *cry*-null (*cry*^{03}) background, to avoid unspecific effects. Experiments involving different GAL4 drivers were performed using eNpHR only, to overcome this issue. For each line, eNpHR-YFP expression was qualitatively checked under a fluorescence microscope to ensure detectable expression levels during embryogenesis (at least from the 15th h AEL).

The following strains were crossed: ElaV^{C155}-GAL4;; cry^{03} females crossed to w*; UAS-ChR2$_{H134R}$; cry^{03} males to pan-neuronally overexpress channelrhodopsin. For halorhodopsin, ElaV^{C155}-GAL4;; females, or other drivers, were crossed to UAS-eNpHR::YFP-50C; UAS-eNpHR::YFP-19C, UAS-eNpHR::YFP-34B males.

Optogenetic manipulation of neuronal activity

Mated adult females were allowed to lay eggs on grape agar (Dutscher, Essex, UK) plates at 25°C supplemented with a small amount live yeast paste. To ensure that embryos receive enough retinal, adults were fed with 4 mM *all-trans*-retinal (Sigma-Aldrich, Poole, UK) dissolved in yeast paste twice a day for three days prior to collection. As a negative control, a group of flies was fed in the absence of retinal. Embryos (1-3 hours AEL) were collected and transferred to a fresh grape agar plate. The plate was placed in a humidified atmosphere inside a 25°C incubator and exposed to collimated light from an overhead LED, positioned to a distance of 17 cm from the embryos. LEDs had peak emission at 470 nm (bandwidth 25 nm, irradiance 466 ± 14 nW·cm^{-2}; OptoLED, Cairn Instruments, Kent, UK) or 565 nm (bandwidth 80 nm, 250 ± 10 µW·cm^{-2}; M565L2, Thorlabs, Newton, NJ). Light was pulsed at 1 Hz, using a Grass S48 stimulator (Grass instruments). Lower frequency stimulation (0.1 Hz) produced seizures of reduced duration, but still significantly higher than the control (RT: 135.50 ± 9.16 s *vs*. 62.23 ± 4.59 s, +LED *vs*. –LED, respectively, $p < 0.001$, $n = 30$), and stimulation at 0.01 Hz was ineffective (RT: 87.70 ± 4.62 s, $n = 30$). The duration of light pulses was based on their ability to evoke or silence neuronal activity. Electrophysiological recordings from aCC/RP2 motoneurons, from newly-eclosed first instar larvae (L1, ~21 hour AEL), determined that short duration pulses (100 ms) were sufficient to ensure photo-activation of both ChR and eNpHR (Figure S2A and S2B). In neurons overexpressing eNpHR, instead of AP inhibition we observed a sustained

rebound firing activity due to anodal-break excitation following the 100 ms light pulses, which has also been indicated in other models [S19]. In this case, longer duration pulses (600 ms) were found to be effective in preventing spike firing.

Embryos were optically treated for a pre-determined time period during embryogenesis (from the 11th to the 19th hour AEL), if not stated otherwise. Shorter periods (2-4 hours) have been used in temporally-controlled experiments to define the presence of a critical period. After manipulation, embryos were transferred into food bottles and maintained at 25°C in complete darkness until ~4 days later when wall climbing L3 were collected and tested for seizure-like behavior. Development was prolonged by lowering the incubation temperature [S20]. Hence, after optical manipulation, newly-hatched L1 were transferred into food bottles and maintained at 18°C until ~9 days later.

Seizure induction in third instar larvae

L3 (wandering stage) were transferred to a plastic dish after washing to remove food residue and gently dried using paper tissue. Once normal crawling behavior resumed, a conductive probe, composed of two tungsten wires (0.1 mm diameter, ~1-2 mm apart) was positioned over the approximate position of the CNS, on the anterior-dorsal cuticle of the animal. A 30 V DC pulse for 3 s, generated by a Grass S88 stimulator (Grass instruments, RI, USA) was applied. In response to the electric stimulus, we observed a transitory paralysis status in which larvae were tonically contracted and, occasionally, exhibited spasms. The time to resumption of normal crawling behavior was measured as recovery time (RT). Normal crawling was defined as a whole body peristaltic wave resulting in forward movement [S21]. Electrical stimulation elicits seizures in both wild-type (WT) and bs-mutant larvae, although the measured RT is significantly longer in the latter, indicative of increased seizure severity. To assess seizure susceptibility four different stimulus-intensity levels (5 – 10 – 20 – 30 V for 3 s) were applied to different sets of larvae. In temperature-controlled experiments, L3 maintained at 18°C during larval development were incubated at 25°C for at least 2 hours before being electroshocked in order to minimize unspecific effects of temperature on locomotion.

Seizure induction in adult flies

To estimate bang-sensitivity, adult flies (1-2 days post-eclosion) were anesthetized by CO_2, placed in empty plastic fly vials (10 flies/vial), and allowed to recover for at least 2 hours. A 10 s mechanical stimulation was delivered via a bench-top vortex (Vortex genie 2, Scientific Industries, Bohemia, FL). The time required for each fly to stand upright after paralysis was measured and a mean value calculated for each vial (RT).

Drug feeding of adult flies

Antiepileptic, i.e. phenytoin (0.4 mg/ml) or gabapentin (0.1 mg/ml), dissolved in DMSO, which has no effect on RT [S21], were added to live yeast paste. Mated adult females were allowed to feed twice a day, for three days, prior to embryo collection. Embryos were collected as previously described and transferred to a nondrug-containing dish for optical stimulation. Previous radio-labeling experiments showed that ingested drug is transferred from gravid

females to oocytes, but is not detected at L3 [S21]. Therefore a direct effect of these drugs on the electroshock response is excluded.

In order to generate a drug-induced seizure model, picrotoxin (0.25 mg/ml), a proconvulsant drug widely used in several animal models including flies [S22-28], was administered to adult gravid females using the same feeding protocol. Exposure of adults to this amount of PTx resulted in a lethality rate of ~10% per day (*vs*. 2% in the -PTx group) and adults exhibit spontaneous seizure behavior ~2 days after first exposure. Nonetheless, egg-laying was not dramatically reduced allowing us to collect sufficient eggs (100-200 per day from 100-150 adults) for 3 consecutive days. To verify that embryos were effectively exposed to PTx, synaptic inputs were recorded from aCC/RP2 motoneurons in newly-hatched L1 (~21 hours AEL, for further details, see the electrophysiology section). We observed a statistically significant increase in synaptic input duration and a decrease in input frequency, as described for a genetic seizure model (see Figure 3). Moreover, 3 out of 10 recordings showed abnormally large synaptic events (duration: 32.59 ± 7.66 s). Such events were never seen in recordings from WT. These data clearly indicate that the embryonic neural network has been exposed to PTx and that the concentration of drug transferred from gravid female to egg is sufficient to perturb embryonic neural circuit activity.

Drugs were obtained from Sigma (UK).

Electrophysiology

Whole-cell voltage- and current-clamp recordings were achieved using thick-walled borosilicate glass electrodes (GC100F-10, Harvard Apparatus, Edenbridge, UK) fire polished to resistances of 15-20 MΩ (L1) and 10-15 MΩ (L3). Recordings were made using a Multiclamp 700B amplifier controlled by pCLAMP (version 10.4) via a Digidata 1440A analog-to-digital converter (Molecular Devices, Sunnyvale, CA). Traces were sampled at 20 kHz and filtered online at 10 kHz. External saline composition was as follows: 135 mM NaCl, 5 mM KCl, 4 mM $MgCl_2 \cdot 6H_2O$, 2 mM $CaCl_2 \cdot 2H_2O$, 5 mM TES and 36 mM sucrose, pH 7.15. Current clamp recordings were performed in the presence of 1 mM mecamylamine to block endogenous cholinergic synaptic currents. Internal patch solution was as follows: 140 mM K^+-D-gluconate, 2 mM $MgCl_2 \cdot 6H_2O$, 2 mM EGTA, 5 mM KCl, and 20 mM HEPES, pH 7.4. KCl and $CaCl_2$ were purchased from Fisher Scientific (Loughborough, UK); sucrose was obtained from BDH (Poole, UK); all remaining chemicals were purchased from Sigma.

Membrane potential changes induced by photo-activation of either ChR, or eNpHR were recorded in current-clamp mode from L1 aCC/RP2 motoneurons held at -60 mV. A 470 nm LED (OptoLED, Cairn Instruments, Kent, UK) and 590 nm LED (M565L2, Thorlabs, Newton, NJ) were connected to an Olympus BX51WI microscope. During recording, light was pulsed onto the sample for 100 or 600 ms at 1 Hz triggered by TTL signals from pClamp (Molecular Devices) to the LED controller.

Spontaneous rhythmic currents (SRCs) were recorded from L3 aCC/RP2 motoneurons for 3 minutes. Traces were sampled at 20 kHz and filtered at 0.2 kHz low pass. Cells with input resistance <0.5 GΩ were not considered for analysis. Synaptic current parameters were examined for each recorded cell using Clampfit (version 10.4). To measure the amplitude of SRCs, the change from baseline to peak current amplitude was determined [S29]. Currents shown were

normalized for cell capacitance (determined by integrating the area under the capacity transient resulting from a step protocol from -60 to -90 mV). The duration of each synaptic event was defined as the time from current initiation until the return to baseline.

Membrane excitability was determined as the number of APs evoked by a series of rectangular depolarizing current pulses (4 pA steps/0.5 s). L3 aCC/RP2 motoneurons were recorded in current-clamp mode and held at -60 mV before the start of the protocol. The mean number of evoked action potentials elicited by incremental current injections was counted for each event. The input-output relationship was evaluated by linear fitting (0-76 pA and 152-228 pA) using GraphPad Prism version 6 (GraphPad Software, San Diego, CA).

Locomotion assay

L3 (wandering stage) were picked from food vials, washed 3-4 times and allowed to crawl at room temperature (20-22°C) for approximately 2 minutes on 140 mm Petri dishes (Thermo Scientific, Loughborough, UK) containing 1% of agarose w/w (SeaKem LE Agarose, Lonza, USA) in deionized water. Locomotion behavior was recorded for 3 minutes with a USB uEye camera (model UI-2230SE-C-HQ, UDS, Germany), at a frame rate of 4 frames/s, using a light panel (model LP812 Jessop, UK) to illuminate the visual field. Larval movement was analyzed using Image-Pro Plus (version 6.3, MediaCybernetics, USA) tracking software and the total path length determined based on larval centroid body measurements. The intensity range used by the software to identify objects of interest was adjusted manually for each larva to achieve optimal contrast.

Statistics

Data were expressed as means ± SEM. Statistical analysis was performed using GraphPad Prism version 6 (GraphPad Software, San Diego, CA) or Origin Pro 9.0 (OriginLab Corp., Northampton, MA). Sample size (n) is reported in each bar and in the tables. Statistical significance between group means was assessed using Student's t-test or ANOVA followed by the Bonferroni's post-hoc test. Significance levels were set at $p < 0.05$.

SUPPLEMENTAL REFERENCES

S1. Lin, D.M., and Goodman, C.S. (1994). Ectopic and increased expression of Fasciclin II alters motoneuron growth cone guidance. Neuron *13*, 507-523.

S2. Salvaterra, P.M., and Kitamoto, T. (2001). Drosophila cholinergic neurons and processes visualized with Gal4/UAS-GFP. Brain Res Gene Expr Patterns *1*, 73-82.

S3. Fei, H., Chow, D.M., Chen, A., Romero-Calderon, R., Ong, W.S., Ackerson, L.C., Maidment, N.T., Simpson, J.H., Frye, M.A., and Krantz, D.E. (2010). Mutation of the Drosophila vesicular GABA transporter disrupts visual figure detection. J Exp Biol *213*, 1717-1730.

S4. Friggi-Grelin, F., Coulom, H., Meller, M., Gomez, D., Hirsh, J., and Birman, S. (2003). Targeted gene expression in Drosophila dopaminergic cells using regulatory sequences from tyrosine hydroxylase. J Neurobiol *54*, 618-627.

S5. Alekseyenko, O.V., Lee, C., and Kravitz, E.A. (2010). Targeted manipulation of serotonergic neurotransmission affects the escalation of aggression in adult male Drosophila melanogaster. PLoS ONE *5*, e10806.

S6. Mahr, A., and Aberle, H. (2006). The expression pattern of the Drosophila vesicular glutamate transporter: a marker protein for motoneurons and glutamatergic centers in the brain. Gene Expr Patterns *6*, 299-309.

S7. Aberle, H., Haghighi, A.P., Fetter, R.D., McCabe, B.D., Magalhaes, T.R., and Goodman, C.S. (2002). Wishful thinking encodes a BMP type II receptor that regulates synaptic growth in Drosophila. Neuron *33*, 545-558.

S8. Cole, S.H., Carney, G.E., McClung, C.A., Willard, S.S., Taylor, B.J., and Hirsh, J. (2005). Two functional but noncomplementing Drosophila tyrosine decarboxylase genes: distinct roles for neural tyramine and octopamine in female fertility. J Biol Chem *280*, 14948-14955.

S9. Taghert, P.H., Hewes, R.S., Park, J.H., O'Brien, M.A., Han, M., and Peck, M.E. (2001). Multiple amidated neuropeptides are required for normal circadian locomotor rhythms in Drosophila. J Neurosci *21*, 6673-6686.

S10. Campbell, G., Goring, H., Lin, T., Spana, E., Andersson, S., Doe, C.Q., and Tomlinson, A. (1994). RK2, a glial-specific homeodomain protein required for embryonic nerve cord condensation and viability in Drosophila. Development *120*, 2957-2966.

S11. Hummel, T., Krukkert, K., Roos, J., Davis, G., and Klambt, C. (2000). Drosophila Futsch/22C10 is a MAP1B-like protein required for dendritic and axonal development. Neuron *26*, 357-370.

S12. Picot, M., Cusumano, P., Klarsfeld, A., Ueda, R., and Rouyer, F. (2007). Light activates output from evening neurons and inhibits output from morning neurons in the Drosophila circadian clock. PLoS Biol *5*, e315.

S13. Crisp, S.J., Evers, J.F., and Bate, M. (2011). Endogenous patterns of activity are required for the maturation of a motor network. J Neurosci *31*, 10445-10450.

S14. Marley, R., Giachello, C.N., Scrutton, N.S., Baines, R.A., and Jones, A.R. (2014). Cryptochrome-dependent magnetic field effect on seizure response in Drosophila larvae. Sci Rep *4*, 5799.

S15. Emery, P., Stanewsky, R., Helfrich-Forster, C., Emery-Le, M., Hall, J.C., and Rosbash, M. (2000). Drosophila CRY is a deep brain circadian photoreceptor. Neuron *26*, 493-504.

S16. Klarsfeld, A., Malpel, S., Michard-Vanhee, C., Picot, M., Chelot, E., and Rouyer, F. (2004). Novel features of cryptochrome-mediated photoreception in the brain circadian clock of Drosophila. J Neurosci *24*, 1468-1477.

S17. Nagel, G., Brauner, M., Liewald, J.F., Adeishvili, N., Bamberg, E., and Gottschalk, A. (2005). Light activation of channelrhodopsin-2 in excitable cells of Caenorhabditis elegans triggers rapid behavioral responses. Curr Biol *15*, 2279-2284.

S18. Inada, K., Kohsaka, H., Takasu, E., Matsunaga, T., and Nose, A. (2011). Optical dissection of neural circuits responsible for Drosophila larval locomotion with halorhodopsin. PLoS ONE *6*, e29019.

S19. Arrenberg, A.B., Del Bene, F., and Baier, H. (2009). Optical control of zebrafish behavior with halorhodopsin. Proc Natl Acad Sci USA *106*, 17968-17973.

S20. Pownser, L. (1935). The effects of temperature on the duration of the developmental stages of Drosophila melanogaster. Physiol Zool *8*, 474-520.

S21. Marley, R., and Baines, R.A. (2011). Increased persistent Na^+ current contributes to seizure in the slamdance bang-sensitive Drosophila mutant. J Neurophysiol *106*, 18-29.

S22. De Deyn, P.P., D'Hooge, R., Marescau, B., and Pei, Y.Q. (1992). Chemical models of epilepsy with some reference to their applicability in the development of anticonvulsants. Epilepsy Res *12*, 87-110.

S23. Hamani, C., and Mello, L.E. (2002). Spontaneous recurrent seizures and neuropathology in the chronic phase of the pilocarpine and picrotoxin model epilepsy. Neurol Res *24*, 199-209.

S24. Mackenzie, L., Medvedev, A., Hiscock, J.J., Pope, K.J., and Willoughby, J.O. (2002). Picrotoxin-induced generalised convulsive seizure in rat: changes in regional distribution and frequency of the power of electroencephalogram rhythms. Clin Neurophysiol *113*, 586-596.

S25. Lee, J., and Wu, C.F. (2002). Electroconvulsive seizure behavior in Drosophila: Analysis of the physiological repertoire underlying a stereotyped action pattern in bang-sensitive mutants. J Neurosci *22*, 11065-11079.

S26. Stilwell, G.E., Saraswati, S., Littleton, J.T., and Chouinard, S.W. (2006). Development of a Drosophila seizure model for in vivo high-throughput drug screening. Eur J Neurosci *24*, 2211-2222.

S27. Wong, K., Stewart, A., Gilder, T., Wu, N., Frank, K., Gaikwad, S., Suciu, C., Dileo, J., Utterback, E., Chang, K., et al. (2010). Modeling seizure-related behavioral and endocrine phenotypes in adult zebrafish. Brain Res *1348*, 209-215.

S28. Lin, W.H., Gunay, C., Marley, R., Prinz, A.A., and Baines, R.A. (2012). Activity-dependent alternative splicing increases persistent sodium current and promotes seizure. J Neurosci *32*, 7267-7277.

S29. Stacey, S.M., Muraro, N.I., Peco, E., Labbe, A., Thomas, G.B., Baines, R.A., and van Meyel, D.J. (2010). Drosophila glial glutamate transporter Eaat1 is regulated by fringe-mediated notch signaling and is essential for larval locomotion. J Neurosci *30*, 14446-14457.
