# Supplementary material for: Inappropriate Neural Activity during a Sensitive Period in Embryogenesis Results in Persistent Seizure-like Behavior
Source: Curr Biol. 2015 Nov 16;25(22):2964–8. doi: 10.1016/j.cub.2015.09.040 (PMC4651905; doi:10.1016/j.cub.2015.09.040)
Supplement: Document S2. Article plus Supplemental Information [file mmc2.pdf]

Report

Current Biology

Inappropriate Neural Activity during a Sensitive Period in Embryogenesis Results in Persistent Seizure-like Behavior

Highlights

- Activity manipulation defines a critical period for circuit functionality
- Abnormal activity during the critical period induces seizure
- Early drug intervention prevents seizure occurrence at postembryonic stages
- Seizure behavior correlates with aberrant synaptic excitation of motoneurons

Authors

Carlo N.G. Giachello, Richard A. Baines

Correspondence

richard.baines@manchester.ac.uk

In Brief

Giachello and Baines show that preventing abnormal neuronal activity during a critical period of *Drosophila* embryogenesis in genetic and chemical models of seizure is sufficient to prevent the emergence of seizure behavior.

CrossMark

Giachello & Baines, 2015, Current Biology *25*, 2964–2968
November 16, 2015 ©2015 The Authors
http://dx.doi.org/10.1016/j.cub.2015.09.040

CellPress

CellPress

Current Biology
Report

Inappropriate Neural Activity during a Sensitive Period in Embryogenesis Results in Persistent Seizure-like Behavior

Carlo N.G. Giachello[1] and Richard A. Baines[1,*]
[1]Faculty of Life Sciences, University of Manchester, Manchester M13 9PT, UK
*Correspondence: richard.baines@manchester.ac.uk
http://dx.doi.org/10.1016/j.cub.2015.09.040
This is an open access article under the CC BY license (http://creativecommons.org/licenses/by/4.0/).

SUMMARY

Maturation of neural circuits requires activity-dependent processes that underpin the emergence of appropriate behavior in the adult. It has been proposed that disruption of these events, during specific critical periods when they exert maximal influence, may lead to neurodevelopmental diseases, including epilepsy [1–3]. However, complexity of neurocircuitry, coupled with the lack of information on network formation in mammals, makes it difficult to directly investigate this hypothesis. Alternative models, including the fruit fly *Drosophila melanogaster*, show remarkable similarities between experimental seizure-like activity and clinical phenotypes [4–6]. In particular, a group of flies, termed bang-sensitive (bs) mutants have been extensively used to investigate the pathophysiological mechanisms underlying seizure [7–12]. Seizure phenotype can be measured in larval stages using an electroshock assay, and this behavior in bs mutants is dramatically reduced following ingestion of typical anti-epileptic drugs (AEDs; [13]). In this study we describe a critical period of embryonic development in *Drosophila* during which manipulation of neural activity is sufficient to significantly influence seizure behavior at postembryonic stages. We show that inhibition of elevated activity, characteristic of bs seizure models, during the critical period is sufficient to suppress seizure. By contrast, increasing neuronal excitation during the same period in wild-type (WT) is sufficient to permanently induce a seizure behavior. Further, we show that induction of seizure in WT correlates with functional alteration of motoneuron inputs that is a characteristic of bs mutants. Induction of seizure is rescued by prior administration of AEDs, opening a new perspective for early drug intervention in the treatment of genetic epilepsy.

RESULTS

Inhibiting Neuronal Activity during Embryogenesis Prevents the Development of a Seizure Phenotype

We have previously shown that the presence of anti-epileptic drugs (AEDs), administered to developing embryos by feeding gravid females, is sufficient to prevent the emergence of seizure behavior in response to electroshock in mature bang-sensitive (bs) larvae (when AEDs are no longer detectable) [13]. We postulated that increased levels of excitatory synaptic activity observed in the CNS of bs mutant embryos are prevented in the presence of AEDs. Manipulation of early neural activity may, therefore, represent a route to control epileptogenesis. In support of this hypothesis, we find a direct correlation between temperature during embryogenesis and subsequent seizure duration in the *bang-senseless* (*bss*; [8, 14]) mutant (Figure S1). This suggests that temperature-sensitive events, for example, neuronal metabolism and/or circuit activity, are crucial during embryogenesis in determining seizure behavior.

To specifically investigate the contribution of neuronal activity, we used optogenetic tools halorhodopsin (eNpHR) and channelrhodopsin (ChR) to selectively modulate neuronal activity during embryogenesis. Whole-cell patch recordings from first instar larvae (L1) confirmed that ChR can depolarize *Drosophila* motoneurons to fire action potentials (APs; Figure S2A), as previously described [15]. Conversely, we found that the effect of eNpHR is stimulation time dependent (Figure S2B; see Supplemental Experimental Procedures). Brief stimulation (λ565 nm, 100 ms/1 Hz) induced a post-inhibitory rebound with significant AP firing (thus could be considered excitatory). Prolonged stimulation (λ565 nm, 600 ms/1 Hz) produced only inhibition of neuronal activity (considered to be inhibitory).

To manipulate neuronal activity in *bss* embryos, we expressed eNpHR in cholinergic neurons, which provide the primary excitatory synaptic drive in insect CNS. Embryos were collected and exposed to light between 11 and 19 hr after egg laying (AEL), and the resulting third instar larvae (L3) were electroshocked 4 days later (Figure 1A). We found that increasing neuronal inhibition with prolonged stimulation (λ565 nm, 600 ms/1 Hz) produced an almost total rescue of seizure behavior (+LED_{600}, Figure 1B), with recovery times

CrossMark

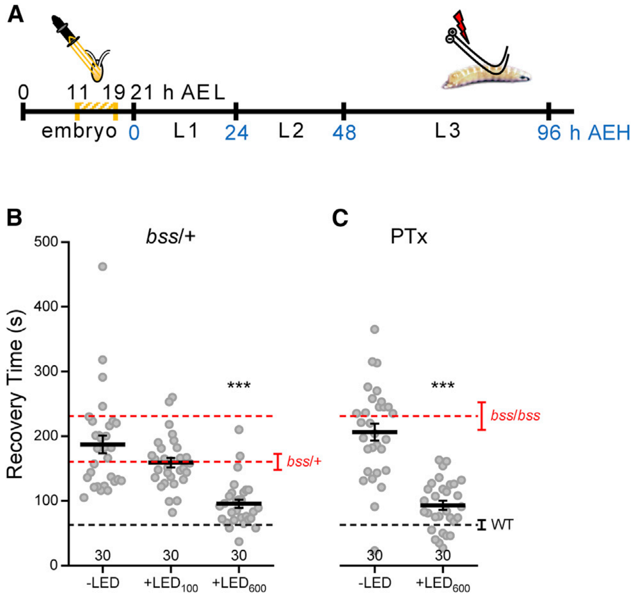

Figure 1. Preventing Neuronal Hyperexcitability during Embryogenesis Is Sufficient to Suppress Seizure

(A) Schematic representation of the experimental procedure. Embryos expressed eNpHR in cholinergic neurons and were exposed to light (λ565 nm, 100 or 600 ms/1 Hz) between 11 and 19 hr AEL. Subsequent L3 larvae were tested for seizure behavior by electroshock. AEH, after embryo hatching.

(B) Electroshock-induced seizure recovery time is significantly reduced in a genetic (*bss*) seizure mutant (*bss*/+; B19-GAL4/eNpHR) following inhibition of neural activity (600-ms light pulses) during embryogenesis (compare +LED_{600} to −LED). By contrast, short duration light pulses of 100 ms (+LED_{100}), which are excitatory (see Figure S2), are without effect. The *bss* mutation is semidominant, conferring a weaker but still significant seizure phenotype in heterozygous progeny (*bss*/+).

(C) Inhibition of neural activity in embryos exposed to the proconvulsant PTx is sufficient to significantly prevent seizure (compare +LED_{600} to −LED).

Data are represented as mean ± SEM. ***$p < 0.001$, Bonferroni's post hoc test. Red and black dotted lines represent reference RTs (mean ± SEM) obtained from *bss*, *bss*/+ and WT, respectively.

(RTs) significantly reduced to values typical of wild-type (WT). Conversely, shorter light pulses that elicit excitation (λ565 nm, 100 ms/1 Hz) had no effect on seizure phenotype (+LED_{100}, Figure 1B). We repeated eNpHR-mediated manipulation in a drug-induced seizure model. Picrotoxin (PTx), a known proconvulsant, produces seizures when administered to *Drosophila* larvae [16, 17], or adults [18]. PTx exposure in WT embryos is sufficient to induce a seizure phenotype, mirroring the effect of the *bss* mutation (−LED, Figure 1C). This effect of PTx was prevented by activation of eNpHR (λ565 nm, 600 ms/1 Hz) during the embryonic critical period (+LED_{600}, Figure 1C). Our manipulations of neural activity during embryogenesis were limited to effects in subsequent larvae (Table S1). Adult flies derived from manipulated embryos show no obvious differences to their non-manipulated counterparts. This is expected and, indeed, is an important control. A lack of carryover to the adult is almost certainly because during metamorphosis (i.e., larva to adult transition), the larval CNS undergoes profound remodeling, involving a significant second wave of neurogenesis and de novo neural circuit formation.

Manipulation of Neuronal Activity during a Critical Period in WT Is Sufficient to Confer a Seizure Phenotype at Postembryonic Stages

The rescue of seizure in L3 that we observed by eNpHR-driven inhibition during embryonic neurogenesis is indicative that neural circuit function can be altered by abnormal levels of synaptic excitation that occur during embryogenesis. A powerful test of this is to increase neural activity in WT embryos and assess for increased seizure behavior at L3. Thus, we expressed ChR pan-neuronally in WT embryos and exposed them to blue light (λ470 nm, 100 ms/1 Hz) between 11 and 19 hr AEL. L3 derived from treated embryos exhibited a marked increase in RT to electroshock (Figure 2A), reaching values comparable with those observed in *bss*. Lower frequency stimulation (0.1 Hz) produced seizures of reduced duration, and stimulation at 0.01 Hz was ineffective (see Supplemental Experimental Procedures). The effect to seizure is, moreover, prevented by feeding the AEDs phenytoin (Phy) or gabapentin (Gbp) to gravid adult females that produced the embryos (Figure 2A). We also tested the effect of increased neuronal inhibition during embryogenesis by expression and activation of eNpHR (λ565 nm, 600 ms/1 Hz) in WT embryos. Surprisingly, subsequent L3 showed an identical heightened RT to electroshock (Figure 2B). Seizure threshold was assessed by applying electroshock stimuli at increasing voltages (from 5 to 30 V, Figure 2C). The *bss* strain exhibits a clear reduction in threshold [8, 19]. A similar reduced threshold was observed in WT L3 optogenetically treated during embryogenesis, suggesting an increase in seizure susceptibility. Taken together, these data suggest that disturbance of normal activity patterns during embryogenesis, rather than just increased excitation, is sufficient to produce a reduced threshold and increased duration of seizure in postembryonic larvae.

In order to test how permanent the alteration to circuit function is, we took advantage of the fact that larval development of *Drosophila* is temperature dependent [20]. Newly hatched L1 were collected after optical manipulation during embryogenesis at 25°C and maintained at either 25°C (4 days) or 18°C (9 days) until the L3 wandering stage. We measured comparable RT values in L3 at both 4 and 9 days, respectively, indicating that this induced seizure behavior is independent of development time and persists through the larval stage (Figure 2D). Optogenetics also facilitates the determination of critical periods. To define a critical period of sensitivity for the effect of altered neural activity with regard to seizure, we performed temporally controlled experiments. We found that perturbing neural activity between 17 and 19 hr AEL is optimal to destabilize CNS function, resulting in a significant increase in RT when tested at L3 (Figure 2E). Again, this effect was limited to L3 and did not carry over to the adult stage (Table S1). Identical results were observed with eNpHR (data not shown).

A significant goal toward a better understanding of epileptogenesis is the determination of whether abnormal activity of neuron number or neuron type is important. To address this, we utilized a range of neuron-specific GAL4 drivers to express eNpHR in the embryonic CNS. Short light pulses (λ565 nm, 100 ms/1 Hz, Figure S2B) were applied in order to trigger excitatory firing to a similar extent to ChR (exposure to a yellow instead of blue light does not require the use of a *cry* null background; see Supplemental Experimental Procedures). Although

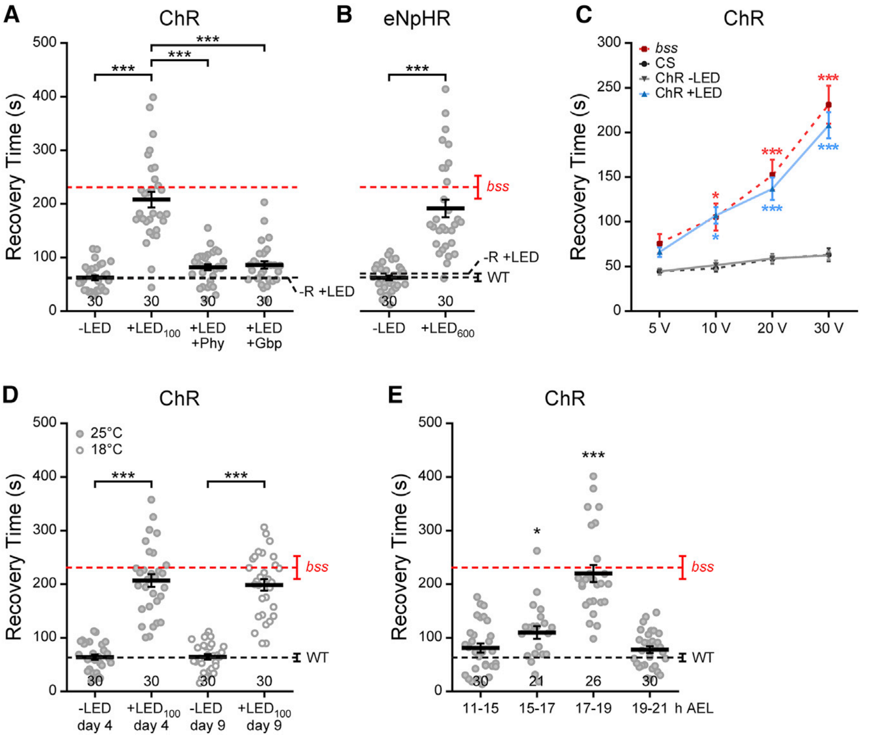

Figure 2. A Critical Period to Influence Seizure

(A and B) RTs measured from L3 that pan-neuronally expressed either ChR (λ470 nm, 100 ms/1 Hz) or eNpHR (λ565 nm, 600 ms/1 Hz) and exposed to light during 11–19 hr AEL of embryogenesis. In both manipulations, disturbing neural activity (+LED) is sufficient to increase the RT of L3 in response to electroshock. Controls were not exposed to light (−LED). The presence of AEDs, Phy (0.4 mg/ml) and Gbp (0.1 mg/ml), during embryogenesis prevents the induction of a seizure phenotype. In order to exclude an unspecific effect of the LED stimulation, embryos were optically manipulated in absence of *all-trans*-retinal (−R+LED, black dotted lines). The RT (mean ± SEM) for homozygous *bss* is shown for reference (red dotted lines).

(C) Seizure threshold is lower in L3 derived from manipulated embryos. Seizure response to varying voltages shows a lower threshold for *bss* [8, 19]. Similarly, larvae in which activity was manipulated during embryogenesis (+LED) require a lower voltage to exhibit a significant increase in RT compared to controls (−LED and WT, n = 20 in each group).

(D) The effect of ChR activation is independent of developmental time. To extend duration of larval stage, larvae were maintained at either 25°C (4 days) or 18°C (9 days) until L3. Two-way ANOVA shows a significant effect of LED treatment ($F_{(1,116)}$ = 249.39, $p < 0.001$), but no effect for developmental time ($F_{(1,116)}$ = 0.18, p = 0.67).

(E) Temporally controlled experiments indicate that manipulation of neuronal activity (ChR, λ470 nm, 100 ms/1 Hz) between 17 and 19 hr AEL is sufficient to induce maximal seizure duration at L3.

Data (A–E) are represented as mean ± SEM. *$p < 0.05$ and ***$p < 0.001$, Bonferroni's post hoc test. The number of tested larvae is shown above the x axis. Red and black dotted lines represent reference RTs (mean ± SEM) obtained from homozygous *bss* and WT, respectively.

the response of different cell types to optogenetic manipulation is problematic to accurately predict, our results implicate a major involvement of both sensory and interneuron components of the larval CNS rather than a contribution by motoneurons (for details, see Table S2). Using a subtractive approach (ElaV^{C155}-GAL4; tsh-GAL80), we tested the relative contribution of all neurons in the brain lobes compared to those in the ventral nerve cord (VNC). Manipulation of the former was not able to produce a bs phenotype when tested in WT L3, indicative that seizures arise following manipulation of the locomotor circuits located in the VNC. Finally, no effects were observed when expression was limited to glia (Table S2). We conclude that efficacy of effect is not overtly dependent on the number of manipulated neurons but rather on their specific connectivity and/or function.

Seizure Phenotype Correlates with Aberrant Synaptic Excitation

A plausible mechanism for prolonged duration of seizure recovery is an increased, and/or uncoordinated, synaptic excitation of larval motoneurons. The dorsal motoneurons, aCC and RP2, receive identical cholinergic excitatory synaptic inputs [21] termed spontaneous rhythmic currents (SRCs; Figure 3) [22]. Compared to WT, SRCs recorded in the bs mutants *slamdance* (*sda*; [13]) and *bss* (Figure 3C) were increased in both amplitude and, in particular, duration. By contrast SRC frequency is dramatically reduced. SRCs recorded from aCC/RP2 in L3 derived from manipulated embryos pan-neuronally expressing ChR (ElaV^{C155}-GAL4) similarly exhibited SRCs with longer duration (Figure 3D) and decreased frequency (Figure 3E). SRC amplitudes, while larger, were not statistically different (Figure 3F). Changes to both duration and frequency were completely reversed by early administration of Phy prior to optogenetic manipulation, by feeding drug to gravid females. Electrophysiological recordings from newly hatched WT L1 derived from adult females fed PTx showed a similar increase in SRC duration and a decrease in frequency with no alteration in amplitude (Figures 3G–3K). Thus, the changes to network activity induced by optogenetic manipulation of the CNS of developing WT embryos results in modifications to neuronal and network properties that are characteristic of bs mutants, or PTx-exposure. Early drug intervention shows that this alteration must occur at a defined period for a heightened seizure phenotype to occur postembryonically. However, it should be noted that we record from only two motoneurons, and how synaptic excitation of the other motoneurons in the larval CNS is affected remains to be determined.

The observed changes to SRC kinetics may be diagnostic of an inability to confine activity levels within the locomotor circuit, consistent with an inability of activity-dependent homeostasis to fully constrain activity levels in this circuit. To determine whether homeostasis is operative under these conditions, we analyzed firing responses of aCC/RP2 in response to injection of depolarizing current. These revealed reduced membrane excitability

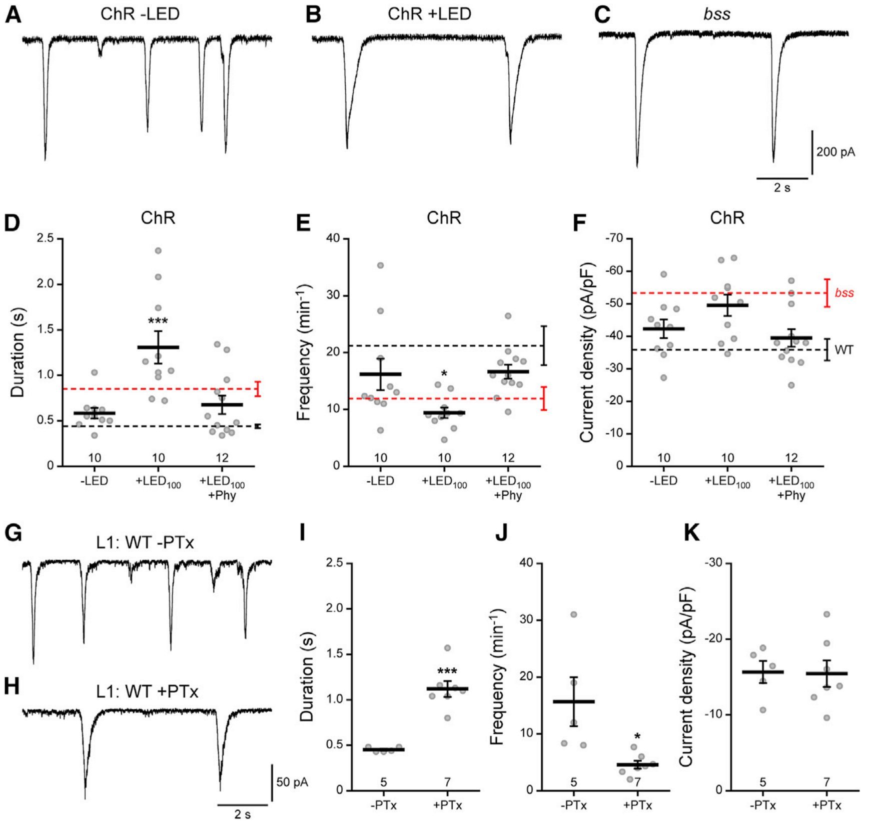

Figure 3. Manipulation of Neuronal Activity Results in an Increase in Synaptic Excitation

(A–C) Whole-cell patch recordings of SRCs from identified L3 aCC/RP2 motoneurons. L3 were derived from WT embryos in which ChR was expressed pan-neuronally (B, ChR+LED) and activated between 11 and 19 hr AEL (λ470 nm, 100 ms/1 Hz). Controls shown were not exposed to light (A, ChR-LED) or are from *bss* L3 (C).

(D and E) Optogenetic manipulation of embryos in which ChR was activated (+LED) shows SRCs at L3 that are increased in duration and reduced in frequency to mirror values recorded in homozygous *bss*. Exposure to Phy during embryogenesis is sufficient to block change to SRC duration and frequency, showing values comparable to those obtained from WT.

(F) SRC amplitudes, normalized to cell capacitance, are not statistically different.

(G and H) Whole-cell patch recordings of SRCs from identified L1 aCC/RP2 motoneurons. L1 were derived from WT embryos exposed to PTx by feeding gravid adult females.

(I and J) PTx exposure during embryogenesis produces a statistically significant increase in synaptic input duration and a decrease in input frequency, as described for *bss*.

(K) SRC amplitudes, normalized to cell capacitance, are not statistically different.

Data (D–K) are represented as mean ± SEM. *$p < 0.05$ and ***$p < 0.001$, Bonferroni's post hoc test. The number of tested larvae is indicated above the x axis. Red and black dotted lines (D–F) represent reference RTs (mean ± SEM) obtained in *bss* and WT, respectively.

(Figure S3), similar to the reduction previously reported in *sda* [13]. Thus, we conclude that while homeostasis, at least in motoneurons, is active, it cannot fully compensate for the change to network excitation that occurs due to activity manipulation during the embryonic critical period.

DISCUSSION

Currently available treatments for seizure are inadequate. AEDs alleviate seizure occurrence and severity but offer no cure. Recently, optogenetic tools have been successfully employed in rodents to inhibit epileptiform activity both in slice [23, 24] and in vivo [25, 26]. Promising results have been achieved by combining eNpHR with on-line seizure detection in order to reduce seizure in an on-demand fashion [27–30]. However, these technologies are still antiepileptic and do not modify the underlying disease-causing mechanisms (i.e., antiepileptogenic). Here, we present evidence that a time-controlled intervention may prevent the onset of seizure, defining a critical period where disturbance of neuronal activity manifests in a heightened response to electroshock. This period coincides precisely with the time window where locomotor circuits are first functional. APs in motoneurons and coordinated body-wall muscle movements first appear at 17 hr AEL [31]. This period has also been determined sensitive for maturation of coordinated motor function in *Drosophila* embryos [32]. Nonetheless, a big challenge still remains to translate this discovery into models evolutionarily closer to humans. In placental mammals, less is known about the time course of neurogenesis occurring during prenatal development. We speculate that in newly formed neuronal networks, there is a time window during which neurons integrate into circuitry and use endogenous activity to refine their properties (i.e., intrinsic excitability, synaptic strength, balance between excitatory and inhibitory synapses, etc.).

It is well established that *Drosophila* motoneurons, like their mammalian counterparts, are able to homeostatically adjust intrinsic excitability to compensate for changing levels of synaptic excitation, in this case from cholinergic presynaptic interneurons [21, 33]. Our results are consistent with a failure of the activity-dependent processes required to incorporate homeostatic limits, based on the dynamic range of activity to which neurons are exposed, during that period. Further investigation of this period may offer the exciting possibility to uncover key molecular components required to define a homeostatic set point. Once set, these limits are seemingly permanent and likely provide the upper and lower extremes for ongoing homeostatic mechanisms that operate in the mature CNS. Capping of activity, with AEDs or optogenetics, during the critical period is sufficient to prevent the emergence of a seizure phenotype in characterized seizure models. If conserved across species, the presence of a critical period in the later stages of neurogenesis could be exploited for therapeutic purposes in humans.

SUPPLEMENTAL INFORMATION

Supplemental Information includes Supplemental Experimental Procedures, three figures, and two tables and can be found with this article online at http://dx.doi.org/10.1016/j.cub.2015.09.040.

AUTHOR CONTRIBUTIONS

C.N.G.G. and R.A.B. designed experiments. C.N.G.G. performed experiments and analyzed data. C.N.G.G. and R.A.B. wrote the paper.

ACKNOWLEDGMENTS

This work was supported by funding from the MRC to R.A.B. (MR/J009180/1) and the Wellcome Trust to C.N.G.G. (097820/Z/11/B). Work on this project benefited from the Manchester Fly Facility, established through funds from the university and the Wellcome Trust (087742/Z/08/Z). The authors thank Miaomiao He and Richard Marley for technical support. We are grateful to members of the R.A.B. group for help and advice during the course of this work. We thank Drs. Stuart Allan, Rob Lucas, Hugh Piggins, Dimitri Kullmann, and Nick Spitzer for critical comments on the manuscript.

Received: April 23, 2015
Revised: August 4, 2015
Accepted: September 16, 2015
Published: November 5, 2015

REFERENCES

1. Doll, C.A., and Broadie, K. (2014). Impaired activity-dependent neural circuit assembly and refinement in autism spectrum disorder genetic models. Front. Cell. Neurosci. *8*, 30.
2. Kroon, T., Sierksma, M.C., and Meredith, R.M. (2013). Investigating mechanisms underlying neurodevelopmental phenotypes of autistic and intellectual disability disorders: a perspective. Front. Syst. Neurosci. *7*, 75.
3. Meredith, R.M., Dawitz, J., and Kramvis, I. (2012). Sensitive time-windows for susceptibility in neurodevelopmental disorders. Trends Neurosci. *35*, 335–344.
4. Baraban, S.C. (2007). Emerging epilepsy models: insights from mice, flies, worms and fish. Curr. Opin. Neurol. *20*, 164–168.
5. Cunliffe, V.T., Baines, R.A., Giachello, C.N., Lin, W.H., Morgan, A., Reuber, M., Russell, C., Walker, M.C., and Williams, R.S. (2015). Epilepsy research methods update: understanding the causes of epileptic seizures and identifying new treatments using non-mammalian model organisms. Seizure *24*, 44–51.
6. Grone, B.P., and Baraban, S.C. (2015). Animal models in epilepsy research: legacies and new directions. Nat. Neurosci. *18*, 339–343.
7. Fergestad, T., Bostwick, B., and Ganetzky, B. (2006). Metabolic disruption in Drosophila bang-sensitive seizure mutants. Genetics *173*, 1357–1364.
8. Parker, L., Padilla, M., Du, Y., Dong, K., and Tanouye, M.A. (2011). Drosophila as a model for epilepsy: bss is a gain-of-function mutation in the para sodium channel gene that leads to seizures. Genetics *187*, 523–534.
9. Sun, L., Gilligan, J., Staber, C., Schutte, R.J., Nguyen, V., O'Dowd, D.K., and Reenan, R. (2012). A knock-in model of human epilepsy in Drosophila reveals a novel cellular mechanism associated with heat-induced seizure. J. Neurosci. *32*, 14145–14155.
10. Stone, B., Evans, L., Coleman, J., and Kuebler, D. (2013). Genetic and pharmacological manipulations that alter metabolism suppress seizure-like activity in Drosophila. Brain Res. *1496*, 94–103.
11. Ehaideb, S.N., Iyengar, A., Ueda, A., Iacobucci, G.J., Cranston, C., Bassuk, A.G., Gubb, D., Axelrod, J.D., Gunawardena, S., Wu, C.F., and Manak, J.R. (2014). prickle modulates microtubule polarity and axonal transport to ameliorate seizures in flies. Proc. Natl. Acad. Sci. USA *111*, 11187–11192.
12. Muraro, N.I., and Baines, R.A. (2008). Drosophila melanogaster in the study of epilepsy. SEB Exp Biol Ser *60*, 141–160.
13. Marley, R., and Baines, R.A. (2011). Increased persistent Na^+ current contributes to seizure in the slamdance bang-sensitive Drosophila mutant. J. Neurophysiol. *106*, 18–29.
14. Ganetzky, B., and Wu, C.F. (1982). Indirect suppression involving behavioral mutants with altered nerve excitability in Drosophila melanogaster. Genetics *100*, 597–614.
15. Zhang, W., Ge, W., and Wang, Z. (2007). A toolbox for light control of Drosophila behaviors through Channelrhodopsin 2-mediated photoactivation of targeted neurons. Eur. J. Neurosci. *26*, 2405–2416.
16. Stilwell, G.E., Saraswati, S., Littleton, J.T., and Chouinard, S.W. (2006). Development of a Drosophila seizure model for in vivo high-throughput drug screening. Eur. J. Neurosci. *24*, 2211–2222.
17. Lin, W.H., Günay, C., Marley, R., Prinz, A.A., and Baines, R.A. (2012). Activity-dependent alternative splicing increases persistent sodium current and promotes seizure. J. Neurosci. *32*, 7267–7277.
18. Lee, J., and Wu, C.F. (2002). Electroconvulsive seizure behavior in Drosophila: analysis of the physiological repertoire underlying a stereotyped action pattern in bang-sensitive mutants. J. Neurosci. *22*, 11065–11079.
19. Kuebler, D., and Tanouye, M.A. (2000). Modifications of seizure susceptibility in Drosophila. J. Neurophysiol. *83*, 998–1009.
20. Pownser, L. (1935). The effects of temperature on the duration of the developmental stages of Drosophila melanogaster. Physiol. Zool. *8*, 474–520.
21. Baines, R.A., Uhler, J.P., Thompson, A., Sweeney, S.T., and Bate, M. (2001). Altered electrical properties in Drosophila neurons developing without synaptic transmission. J. Neurosci. *21*, 1523–1531.
22. Rohrbough, J., and Broadie, K. (2002). Electrophysiological analysis of synaptic transmission in central neurons of Drosophila larvae. J. Neurophysiol. *88*, 847–860.
23. Tønnesen, J., Sørensen, A.T., Deisseroth, K., Lundberg, C., and Kokaia, M. (2009). Optogenetic control of epileptiform activity. Proc. Natl. Acad. Sci. USA *106*, 12162–12167.
24. Berglind, F., Ledri, M., Sørensen, A.T., Nikitidou, L., Melis, M., Bielefeld, P., Kirik, D., Deisseroth, K., Andersson, M., and Kokaia, M. (2014). Optogenetic inhibition of chemically induced hypersynchronized bursting in mice. Neurobiol. Dis. *65*, 133–141.
25. Sukhotinsky, I., Chan, A.M., Ahmed, O.J., Rao, V.R., Gradinaru, V., Ramakrishnan, C., Deisseroth, K., Majewska, A.K., and Cash, S.S. (2013). Optogenetic delay of status epilepticus onset in an in vivo rodent epilepsy model. PLoS ONE *8*, e62013.
26. Chiang, C.C., Ladas, T.P., Gonzalez-Reyes, L.E., and Durand, D.M. (2014). Seizure suppression by high frequency optogenetic stimulation using in vitro and in vivo animal models of epilepsy. Brain Stimulat. *7*, 890–899.
27. Wykes, R.C., Heeroma, J.H., Mantoan, L., Zheng, K., MacDonald, D.C., Deisseroth, K., Hashemi, K.S., Walker, M.C., Schorge, S., and Kullmann, D.M. (2012). Optogenetic and potassium channel gene therapy in a rodent model of focal neocortical epilepsy. Sci. Transl. Med. *4*, 161ra152.
28. Paz, J.T., Davidson, T.J., Frechette, E.S., Delord, B., Parada, I., Peng, K., Deisseroth, K., and Huguenard, J.R. (2013). Closed-loop optogenetic control of thalamus as a tool for interrupting seizures after cortical injury. Nat. Neurosci. *16*, 64–70.
29. Armstrong, C., Krook-Magnuson, E., Oijala, M., and Soltesz, I. (2013). Closed-loop optogenetic intervention in mice. Nat. Protoc. *8*, 1475–1493.
30. Krook-Magnuson, E., Armstrong, C., Oijala, M., and Soltesz, I. (2013). On-demand optogenetic control of spontaneous seizures in temporal lobe epilepsy. Nat. Commun. *4*, 1376.
31. Baines, R.A., and Bate, M. (1998). Electrophysiological development of central neurons in the Drosophila embryo. J. Neurosci. *18*, 4673–4683.
32. Crisp, S.J., Evers, J.F., and Bate, M. (2011). Endogenous patterns of activity are required for the maturation of a motor network. J. Neurosci. *31*, 10445–10450.
33. Baines, R.A. (2003). Postsynaptic protein kinase A reduces neuronal excitability in response to increased synaptic excitation in the Drosophila CNS. J. Neurosci. *23*, 8664–8672.

Current Biology
Supplemental Information

Inappropriate Neural Activity during a Sensitive Period in Embryogenesis Results in Persistent Seizure-like Behavior

Carlo N.G. Giachello and Richard A. Baines

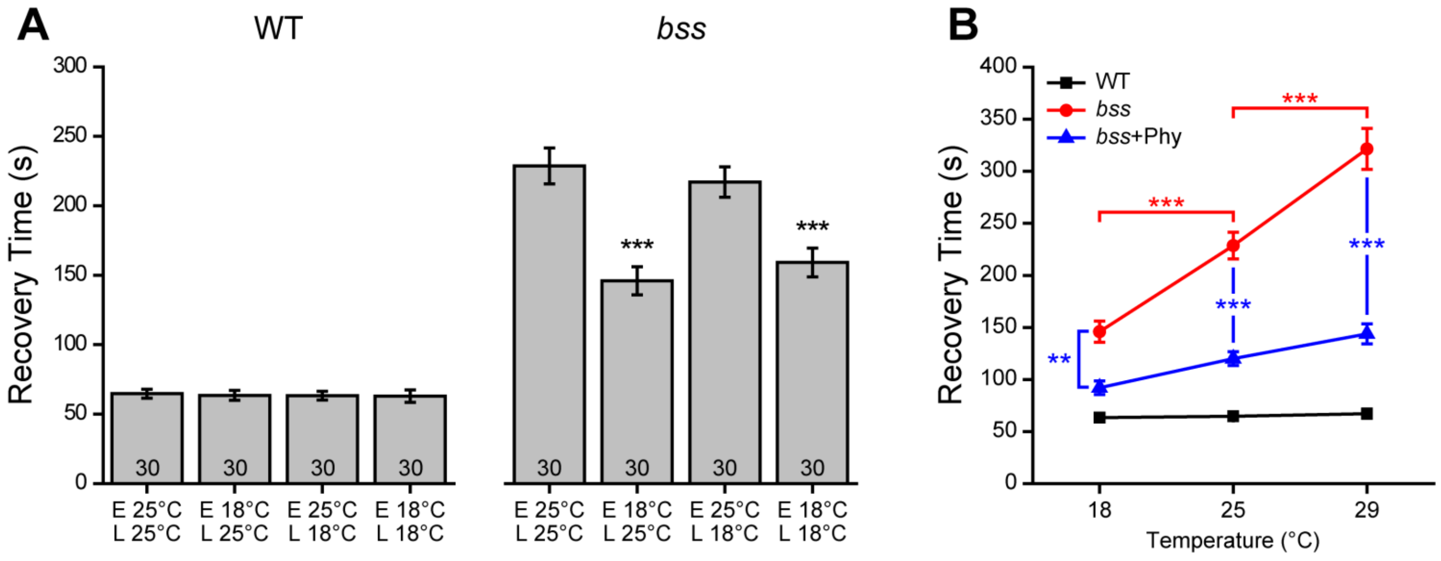

Figure S1. Developmental temperature during embryogenesis affects seizure severity

(**A**) RTs measured at L3 from WT and homozygous *bss* pre-incubated at different temperatures during both embryonic (E) and larval (L) stages. Only *bss* L3 derived from embryos maintained at 18°C, compared to either normal (25°C) or elevated (29°C) temperature, showed a statistically significant recovery of seizure-behavior. By contrast, altering temperature after hatching had no effect on seizure severity. The number of tested larvae is indicated in the bars. (**B**) RTs from WT and homozygous *bss* incubated at different temperatures (18°C, 25°C and 29°C) during embryogenesis only. Newly-hatched L1 from all groups were maintained at 25°C until L3. Only *bss* embryos maintained at 18°C show significantly reduced RT ($F_{(2, 261)} = 223.70$, $p < 0.001$, n = 30 in each group), suggesting that temperature-sensitive events, such as neuronal metabolism and/or circuit activity (see Figure 1B), are implicated in determining seizure-behavior. *bss* larvae treated with phenytoin (Phy) during embryogenesis exhibited a reduced dependency on temperature and RTs were lower with respect to the untreated *bss* line, indicating a beneficial treatment of the drug. To determine AED sensitivity, *bss* embryos were exposed to Phy by feeding gravid adult females. Data (A and B) are represented as mean ± SEM. *** = $p < 0.001$ with respect to the E25°C-L25°C group, Bonferroni's *post-hoc* test.

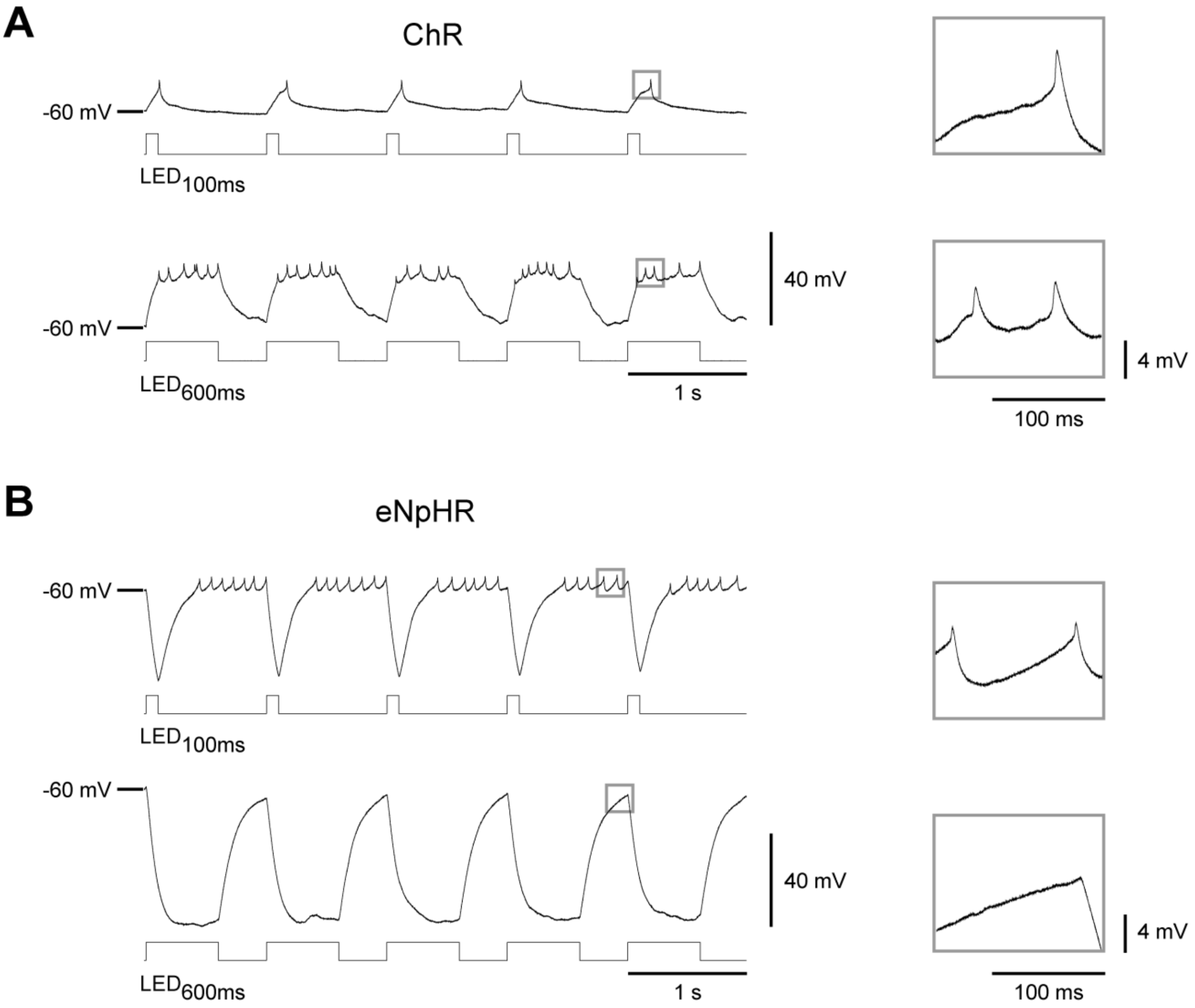

Figure S2. The effect of eNpHR is stimulation-time dependent

(**A**) Whole-cell patch recordings from aCC/RP2 motoneurons in newly-hatched L1 (~21 hours AEL) showing membrane potential changes due to activation of ChR (λ470 nm, 100 or 600 ms/1 Hz). Single (1.37 ± 0.26 APs per light pulse, n = 5) or multiple (4.97 ± 0.56 APs per light pulse, n = 5) action potentials (APs) are elicited following ChR activation for either short (100 ms) or long (600 ms) periods, respectively. (**B**) In eNpHR-expressing neurons short duration (λ565 nm, 100 ms/1 Hz) activation produced brief hyperpolarization events that gave rise to rebound AP firing (4.42 ± 1.34 APs per light pulse, n = 8), due to anodal-break excitation. By contrast, prolonged photo-activation (λ565 nm, 600 ms/1 Hz) produced only inhibitory hyperpolarizing changes to the membrane potential with no rebound firing (0.36 ± 0.31 APs per light pulse, n = 8). Indeed, pulse duration (100 *vs*. 600 ms) affects the ability of eNpHR to suppress seizure in our model (see Figure 1B). Inset shows magnified images of the light-evoked APs.

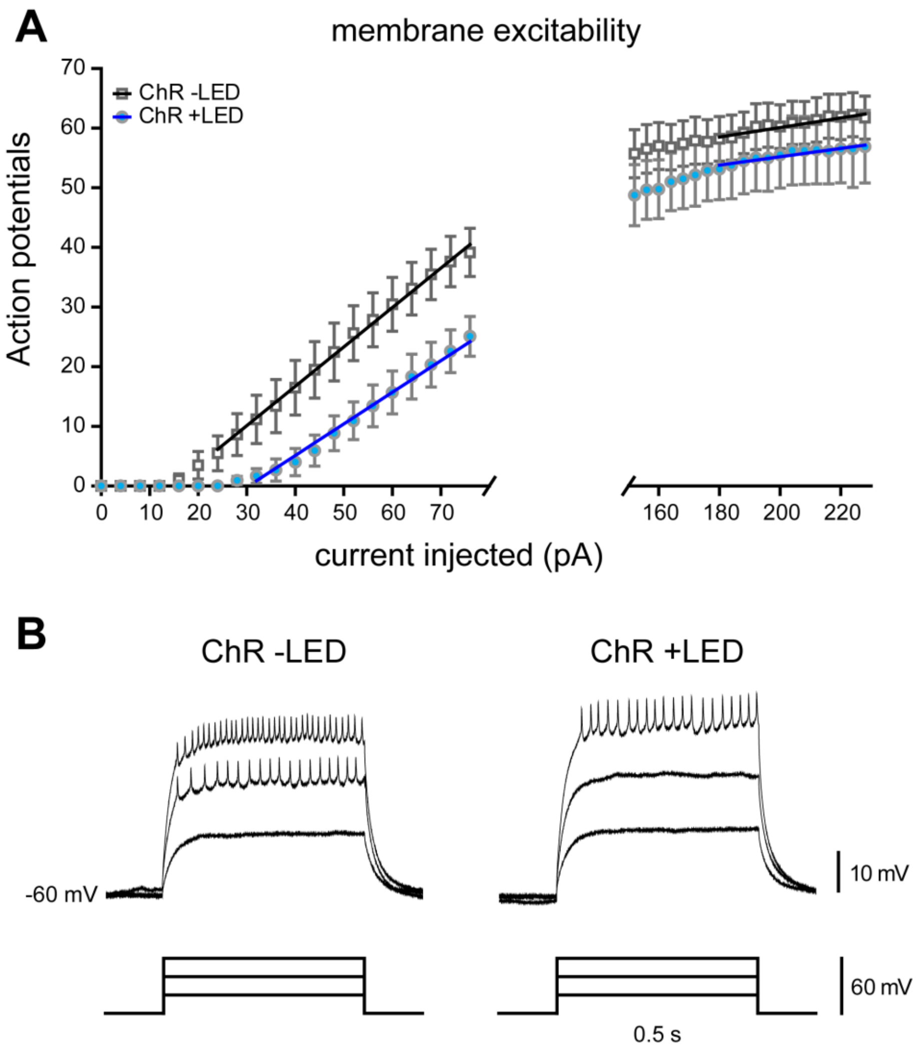

Figure S3. L3 motoneurons derived from optogenetically-manipulated embryos exhibit reduced membrane excitability

(**A**) Changes in membrane excitability of L3 aCC/RP2 motoneurons, following the increased synaptic excitation (see Figure 3), were determined by injection of constant current (4 pA steps/0.5 s). The current-firing relationship was evaluated by linear regression. The linear part (28-76 pA) and the plateau (180-228 pA) of the curve were analyzed separately. The number of APs fired is significantly less in manipulated (+LED, n = 12) compared to control (-LED, n = 13) L3. The fitted lines exhibit similar slope values (linear part: 0.53 ± 0.02 *vs*. 0.66 ± 0.01, +LED *vs*. -LED, respectively; and plateau: 0.07 ± 0.01 *vs*. 0.08 ± 0.01, +LED *vs*. -LED, respectively), but different intercepts considering both the rising (-16.10 ± 0.97 *vs*. -9.69 ± 0.65, +LED *vs*. -LED, respectively, $p < 0.001$) and plateau (41.19 ± 1.62 *vs*. 44.10 ± 1.68, +LED *vs*. -LED, respectively, $p = 0.004$) elements of the curves. No difference in resting membrane potential, input resistance or capacitance was observed (data not shown). Data are represented as mean ± SEM. (**B**) Representative traces showing firing of APs by successively greater depolarizing current injections (20, 40, and 60 pA/0.5 s) in aCC.

Table S1. Neuronal remodeling during pupation resets the effect of optogenetic manipulation.

	Treatment	n	Mean ± SEM	p-value
bss/+	eNpHR -LED	60	77.67 ± 14.89 s	
bss/+	eNpHR +LED_{600ms}	60	67.83 ± 9.43 s	n.s.
bss/+	eNpHR +LED_{100ms}	60	73.50 ± 13.34 s	n.s.
WT + PTx	eNpHR -LED	100	0.73 ± 0.05 s	
WT + PTx	eNpHR +LED_{600ms}	100	0.71 ± 0.06 s	n.s.
WT	ChR -$LED_{11\text{-}15\ h}$	15	0.73 ± 0.14 s	
WT	ChR +$LED_{11\text{-}15\ h}$	18	0.71 ± 0.07 s	n.s.
WT	ChR -$LED_{15\text{-}17\ h}$	41	0.75 ± 0.15 s	
WT	ChR +$LED_{15\text{-}17\ h}$	47	0.73 ± 0.11 s	n.s.
WT	ChR -$LED_{17\text{-}19\ h}$	49	0.77 ± 0.11 s	
WT	ChR +$LED_{17\text{-}19\ h}$	54	0.75 ± 0.07 s	n.s.
WT	ChR -$LED_{19\text{-}21\ h}$	64	0.68 ± 0.11 s	
WT	ChR +$LED_{19\text{-}21\ h}$	41	0.70 ± 0.10 s	n.s.
Reference values				
WT		80	0.70 ± 0.10 s	
bss/bss		100	242.66 ± 25.30 s	
bss/+		90	88.39 ± 28.61 s	

RTs measured by vortexing adult flies, derived from manipulated embryos. (**A**) The ability of eNpHR-activation to rescue seizure in L3 carrying the *bss* mutation, is not maintained in adults flies derived from these larvae (cf. Figure 1B). (**B**) The effect of embryonic exposure to PTx to induce seizure in L3, is also not carried forward to the adult (cf. Figure 1C). This loss of effect is not influenced by activation of eNpHR during embryogenesis. (**C**) Similarly, neuronal remodeling during pupation completely rescues the ChR-induced seizure-phenotype observed in L3 (cf. Figure 2E).

Table S2. Different neuronal subpopulations contribute unequally to seizure predisposition.

GAL4 lines (neuron types)	Treatment	n	Mean ± SEM	p-value
ElaV^{C155}-GAL4 (all neurons) [S1]	-LED	18	70.22 ± 8.72 s	
	+LED$_{100ms}$	25	177.08 ± 16.48 s	<0.001
B19-GAL4 (cholinergic) [S2]	-LED	30	62.73 ± 3.86 s	
	+LED$_{100ms}$	30	187.57 ± 16.86 s	<0.001
vGat-GAL4 (GABAergic) [S3]	-LED	30	60.50 ± 5.19 s	
	+LED$_{100ms}$	30	163.03 ± 12.78 s	<0.001
TH-GAL4 (dopaminergic)[S4]	-LED	30	63.17 ± 3.95 s	
	+LED$_{100ms}$	30	108.93 ± 10.95 s	<0.001
TRH-GAL4 (serotonergic) [S5]	-LED	30	60.37 ± 4.27 s	
	+LED$_{100ms}$	30	100.97 ± 11.45 s	<0.001
OK371-GAL4 (glutamatergic) [S6]	-LED	30	59.87 ± 4.30 s	
	+LED$_{100ms}$	30	123.27 ± 7.73 s	<0.001
OK6-GAL4 (motoneurons) [S7]	-LED	30	62.27 ± 3.98 s	
	+LED$_{100ms}$	30	73.30 ± 4.42 s	n.s.
tdc2-GAL4 (tyraminergic/octopaminergic) [S8]	-LED	30	64.47 ± 3.72 s	
	+LED$_{100ms}$	30	74.17 ± 6.13 s	n.s.
386Y-GAL4 (peptidergic) [S9]	-LED	30	61.87 ± 4.40 s	
	+LED$_{100ms}$	30	93.30 ± 7.20 s	<0.001
Repo-GAL4 (glia cells) [S10]	-LED	30	62.73 ± 3.86 s	
	+LED$_{100ms}$	30	74.83 ± 5.82 s	n.s.
P0163-GAL4 (sensory) [S11]	-LED	30	60.13 ± 4.14 s	
	+LED$_{100ms}$	30	121.50 ± 12.67 s	<0.001
cry-GAL4 (Cry-positive) [S12]	-LED	30	63.83 ± 4.41 s	
	+LED$_{100ms}$	30	87.43 ± 6.87 s	0.005
ElaV^{C155}-GAL4;tsh-GAL80 (brain lobes)	-LED	30	60.67 ± 3.01 s	
	+LED$_{100ms}$	25	52.96 ± 6.64 s	n.s.

RTs of L3 to electroshock derived from embryos in which neuronal activity was manipulated by expression of eNpHR in different neuron types. Embryos were exposed to light (λ565 nm, 100 ms/1 Hz) between 11-19 hours AEL (see Figure 1A). In order to exclude an unspecific effect of the LED stimulation, embryos were also optically manipulated in absence of *all-trans*-retinal

(data not shown). Data were statistically compared to the corresponding -LED group using the Student's *t*-test.

Collectively, these data implicate the major neurotransmitter systems, e.g. cholinergic (B19), GABAergic (vGAT), glutamatergic (OK371), serotonergic (TRH), dopaminergic (TH) and peptidergic (386Y) to contribute to increase seizure duration. By contrast, increased activity in neurons directly involved in locomotion, i.e. motoneurons (OK6) and octopaminergic (tdc2) modulatory neurons was without effect. Motoneurons are glutamatergic and are also labeled by OK371 [S6]. By subtraction, we conclude that the effect of OK371-driven expression is achieved through manipulation of activity of the small population of glutamatergic interneurons (6 for each hemisegment) present in the embryonic CNS [S6]. We also observed increased seizure-like behavior following activity-manipulation of peripheral sensory inputs (P0163), which presumably contribute to levels of excitation centrally and influence the frequency of episodic activity in the developing motor network [S13]. Recent evidence indicates that the activation of Cry, a blue light-sensing photoreceptor, during embryogenesis, is sufficient to increase the seizure-phenotype of *Drosophila* WT L3 [S14]. Hence, we selectively manipulated the activity of Cry-positive neurons, obtaining a mild, but still significant, increase in RT. Just 6 Cry-positive neurons are present in the L3 CNS [S15, 16], indicating that activity-manipulation of even a small subset of neurons is sufficient to give a detectable effect. Finally, using a subtractive approach (ElaV^{C155}-GAL4; tsh-GAL80), we tested the relative contribution of all neurons in the brain lobes compared to those in the ventral nerve cord (VNC). Manipulation of the former was not able to produce a bs-phenotype when tested in WT L3 indicative that seizures arise following manipulation of the locomotor circuits located in the VNC. Finally, no effects were observed when expression was limited to glia (Repo).

SUPPLEMENTAL EXPERIMENTAL PROCEDURES

Fly stocks

Flies were maintained on standard corn meal medium at 25°C. Canton S flies from Bloomington *Drosophila* Stock Center was used as WT. Bang-sensitive flies used in this study were *bang senseless*[1] kindly provided by Kevin O'Dell. The following fly stocks were kindly provided by the respective researchers: channelrhodopsin w*; UAS-ChR2$_{H134R}$ (Stefan Pulver; [S17]), halorhodopsin UAS-eNpHR::YFP-50C; UAS-eNpHR::YFP-19C, UAS-eNpHR::YFP-34B (Akinao Nose; [S18]), B19-GAL4 (Paul M. Salvaterra), vGat-GAL4 (Justin Blau), TH-GAL4 and tdc2-GAL4 (Chris Elliott), OK6-GAL4 (Andreas Prokop), Repo-GAL4 (Angela Giangrande), P0163-GAL4 (Matthias Landgraf), cry-GAL4-19 (Ralf Stanewsky). All other lines were obtained from the Bloomington *Drosophila* Stock Center.

Previously published results indicate that embryonic exposure to blue light is sufficient to increase the RT in third instar larvae (L3) due to the activation of endogenous cryptochrome (Cry) [S14]. In this study, all the experiments involving blue light were performed in a *cry*-null (*cry*^{03}) background, to avoid unspecific effects. Experiments involving different GAL4 drivers were performed using eNpHR only, to overcome this issue. For each line, eNpHR-YFP expression was qualitatively checked under a fluorescence microscope to ensure detectable expression levels during embryogenesis (at least from the 15th h AEL).

The following strains were crossed: ElaV^{C155}-GAL4;; cry^{03} females crossed to w*; UAS-ChR2$_{H134R}$; cry^{03} males to pan-neuronally overexpress channelrhodopsin. For halorhodopsin, ElaV^{C155}-GAL4;; females, or other drivers, were crossed to UAS-eNpHR::YFP-50C; UAS-eNpHR::YFP-19C, UAS-eNpHR::YFP-34B males.

Optogenetic manipulation of neuronal activity

Mated adult females were allowed to lay eggs on grape agar (Dutscher, Essex, UK) plates at 25°C supplemented with a small amount live yeast paste. To ensure that embryos receive enough retinal, adults were fed with 4 mM *all-trans*-retinal (Sigma-Aldrich, Poole, UK) dissolved in yeast paste twice a day for three days prior to collection. As a negative control, a group of flies was fed in the absence of retinal. Embryos (1-3 hours AEL) were collected and transferred to a fresh grape agar plate. The plate was placed in a humidified atmosphere inside a 25°C incubator and exposed to collimated light from an overhead LED, positioned to a distance of 17 cm from the embryos. LEDs had peak emission at 470 nm (bandwidth 25 nm, irradiance 466 ± 14 nW·cm^{-2}; OptoLED, Cairn Instruments, Kent, UK) or 565 nm (bandwidth 80 nm, 250 ± 10 μW·cm^{-2}; M565L2, Thorlabs, Newton, NJ). Light was pulsed at 1 Hz, using a Grass S48 stimulator (Grass instruments). Lower frequency stimulation (0.1 Hz) produced seizures of reduced duration, but still significantly higher than the control (RT: 135.50 ± 9.16 s *vs*. 62.23 ± 4.59 s, +LED *vs*. –LED, respectively, $p < 0.001$, $n = 30$), and stimulation at 0.01 Hz was ineffective (RT: 87.70 ± 4.62 s, $n = 30$). The duration of light pulses was based on their ability to evoke or silence neuronal activity. Electrophysiological recordings from aCC/RP2 motoneurons, from newly-eclosed first instar larvae (L1, ~21 hour AEL), determined that short duration pulses (100 ms) were sufficient to ensure photo-activation of both ChR and eNpHR (Figure S2A and S2B). In neurons overexpressing eNpHR, instead of AP inhibition we observed a sustained

rebound firing activity due to anodal-break excitation following the 100 ms light pulses, which has also been indicated in other models [S19]. In this case, longer duration pulses (600 ms) were found to be effective in preventing spike firing.

Embryos were optically treated for a pre-determined time period during embryogenesis (from the 11th to the 19th hour AEL), if not stated otherwise. Shorter periods (2-4 hours) have been used in temporally-controlled experiments to define the presence of a critical period. After manipulation, embryos were transferred into food bottles and maintained at 25°C in complete darkness until ~4 days later when wall climbing L3 were collected and tested for seizure-like behavior. Development was prolonged by lowering the incubation temperature [S20]. Hence, after optical manipulation, newly-hatched L1 were transferred into food bottles and maintained at 18°C until ~9 days later.

Seizure induction in third instar larvae

L3 (wandering stage) were transferred to a plastic dish after washing to remove food residue and gently dried using paper tissue. Once normal crawling behavior resumed, a conductive probe, composed of two tungsten wires (0.1 mm diameter, ~1-2 mm apart) was positioned over the approximate position of the CNS, on the anterior-dorsal cuticle of the animal. A 30 V DC pulse for 3 s, generated by a Grass S88 stimulator (Grass instruments, RI, USA) was applied. In response to the electric stimulus, we observed a transitory paralysis status in which larvae were tonically contracted and, occasionally, exhibited spasms. The time to resumption of normal crawling behavior was measured as recovery time (RT). Normal crawling was defined as a whole body peristaltic wave resulting in forward movement [S21]. Electrical stimulation elicits seizures in both wild-type (WT) and bs-mutant larvae, although the measured RT is significantly longer in the latter, indicative of increased seizure severity. To assess seizure susceptibility four different stimulus-intensity levels (5 – 10 – 20 – 30 V for 3 s) were applied to different sets of larvae. In temperature-controlled experiments, L3 maintained at 18°C during larval development were incubated at 25°C for at least 2 hours before being electroshocked in order to minimize unspecific effects of temperature on locomotion.

Seizure induction in adult flies

To estimate bang-sensitivity, adult flies (1-2 days post-eclosion) were anesthetized by CO_2, placed in empty plastic fly vials (10 flies/vial), and allowed to recover for at least 2 hours. A 10 s mechanical stimulation was delivered via a bench-top vortex (Vortex genie 2, Scientific Industries, Bohemia, FL). The time required for each fly to stand upright after paralysis was measured and a mean value calculated for each vial (RT).

Drug feeding of adult flies

Antiepileptic, i.e. phenytoin (0.4 mg/ml) or gabapentin (0.1 mg/ml), dissolved in DMSO, which has no effect on RT [S21], were added to live yeast paste. Mated adult females were allowed to feed twice a day, for three days, prior to embryo collection. Embryos were collected as previously described and transferred to a nondrug-containing dish for optical stimulation. Previous radio-labeling experiments showed that ingested drug is transferred from gravid

females to oocytes, but is not detected at L3 [S21]. Therefore a direct effect of these drugs on the electroshock response is excluded.

In order to generate a drug-induced seizure model, picrotoxin (0.25 mg/ml), a proconvulsant drug widely used in several animal models including flies [S22-28], was administered to adult gravid females using the same feeding protocol. Exposure of adults to this amount of PTx resulted in a lethality rate of ~10% per day (*vs*. 2% in the -PTx group) and adults exhibit spontaneous seizure behavior ~2 days after first exposure. Nonetheless, egg-laying was not dramatically reduced allowing us to collect sufficient eggs (100-200 per day from 100-150 adults) for 3 consecutive days. To verify that embryos were effectively exposed to PTx, synaptic inputs were recorded from aCC/RP2 motoneurons in newly-hatched L1 (~21 hours AEL, for further details, see the electrophysiology section). We observed a statistically significant increase in synaptic input duration and a decrease in input frequency, as described for a genetic seizure model (see Figure 3). Moreover, 3 out of 10 recordings showed abnormally large synaptic events (duration: 32.59 ± 7.66 s). Such events were never seen in recordings from WT. These data clearly indicate that the embryonic neural network has been exposed to PTx and that the concentration of drug transferred from gravid female to egg is sufficient to perturb embryonic neural circuit activity.

Drugs were obtained from Sigma (UK).

Electrophysiology

Whole-cell voltage- and current-clamp recordings were achieved using thick-walled borosilicate glass electrodes (GC100F-10, Harvard Apparatus, Edenbridge, UK) fire polished to resistances of 15-20 MΩ (L1) and 10-15 MΩ (L3). Recordings were made using a Multiclamp 700B amplifier controlled by pCLAMP (version 10.4) via a Digidata 1440A analog-to-digital converter (Molecular Devices, Sunnyvale, CA). Traces were sampled at 20 kHz and filtered online at 10 kHz. External saline composition was as follows: 135 mM NaCl, 5 mM KCl, 4 mM $MgCl_2·6H_2O$, 2 mM $CaCl_2·2H_2O$, 5 mM TES and 36 mM sucrose, pH 7.15. Current clamp recordings were performed in the presence of 1 mM mecamylamine to block endogenous cholinergic synaptic currents. Internal patch solution was as follows: 140 mM K^+-D-gluconate, 2 mM $MgCl_2·6H_2O$, 2 mM EGTA, 5 mM KCl, and 20 mM HEPES, pH 7.4. KCl and $CaCl_2$ were purchased from Fisher Scientific (Loughborough, UK); sucrose was obtained from BDH (Poole, UK); all remaining chemicals were purchased from Sigma.

Membrane potential changes induced by photo-activation of either ChR, or eNpHR were recorded in current-clamp mode from L1 aCC/RP2 motoneurons held at -60 mV. A 470 nm LED (OptoLED, Cairn Instruments, Kent, UK) and 590 nm LED (M565L2, Thorlabs, Newton, NJ) were connected to an Olympus BX51WI microscope. During recording, light was pulsed onto the sample for 100 or 600 ms at 1 Hz triggered by TTL signals from pClamp (Molecular Devices) to the LED controller.

Spontaneous rhythmic currents (SRCs) were recorded from L3 aCC/RP2 motoneurons for 3 minutes. Traces were sampled at 20 kHz and filtered at 0.2 kHz low pass. Cells with input resistance <0.5 GΩ were not considered for analysis. Synaptic current parameters were examined for each recorded cell using Clampfit (version 10.4). To measure the amplitude of SRCs, the change from baseline to peak current amplitude was determined [S29]. Currents shown were

normalized for cell capacitance (determined by integrating the area under the capacity transient resulting from a step protocol from -60 to -90 mV). The duration of each synaptic event was defined as the time from current initiation until the return to baseline.

Membrane excitability was determined as the number of APs evoked by a series of rectangular depolarizing current pulses (4 pA steps/0.5 s). L3 aCC/RP2 motoneurons were recorded in current-clamp mode and held at -60 mV before the start of the protocol. The mean number of evoked action potentials elicited by incremental current injections was counted for each event. The input-output relationship was evaluated by linear fitting (0-76 pA and 152-228 pA) using GraphPad Prism version 6 (GraphPad Software, San Diego, CA).

Locomotion assay

L3 (wandering stage) were picked from food vials, washed 3-4 times and allowed to crawl at room temperature (20-22°C) for approximately 2 minutes on 140 mm Petri dishes (Thermo Scientific, Loughborough, UK) containing 1% of agarose w/w (SeaKem LE Agarose, Lonza, USA) in deionized water. Locomotion behavior was recorded for 3 minutes with a USB uEye camera (model UI-2230SE-C-HQ, UDS, Germany), at a frame rate of 4 frames/s, using a light panel (model LP812 Jessop, UK) to illuminate the visual field. Larval movement was analyzed using Image-Pro Plus (version 6.3, MediaCybernetics, USA) tracking software and the total path length determined based on larval centroid body measurements. The intensity range used by the software to identify objects of interest was adjusted manually for each larva to achieve optimal contrast.

Statistics

Data were expressed as means ± SEM. Statistical analysis was performed using GraphPad Prism version 6 (GraphPad Software, San Diego, CA) or Origin Pro 9.0 (OriginLab Corp., Northampton, MA). Sample size (n) is reported in each bar and in the tables. Statistical significance between group means was assessed using Student’s t-test or ANOVA followed by the Bonferroni’s post-hoc test. Significance levels were set at $p < 0.05$.

SUPPLEMENTAL REFERENCES

S1. Lin, D.M., and Goodman, C.S. (1994). Ectopic and increased expression of Fasciclin II alters motoneuron growth cone guidance. Neuron *13*, 507-523.

S2. Salvaterra, P.M., and Kitamoto, T. (2001). Drosophila cholinergic neurons and processes visualized with Gal4/UAS-GFP. Brain Res Gene Expr Patterns *1*, 73-82.

S3. Fei, H., Chow, D.M., Chen, A., Romero-Calderon, R., Ong, W.S., Ackerson, L.C., Maidment, N.T., Simpson, J.H., Frye, M.A., and Krantz, D.E. (2010). Mutation of the Drosophila vesicular GABA transporter disrupts visual figure detection. J Exp Biol *213*, 1717-1730.

S4. Friggi-Grelin, F., Coulom, H., Meller, M., Gomez, D., Hirsh, J., and Birman, S. (2003). Targeted gene expression in Drosophila dopaminergic cells using regulatory sequences from tyrosine hydroxylase. J Neurobiol *54*, 618-627.

S5. Alekseyenko, O.V., Lee, C., and Kravitz, E.A. (2010). Targeted manipulation of serotonergic neurotransmission affects the escalation of aggression in adult male Drosophila melanogaster. PLoS ONE *5*, e10806.

S6. Mahr, A., and Aberle, H. (2006). The expression pattern of the Drosophila vesicular glutamate transporter: a marker protein for motoneurons and glutamatergic centers in the brain. Gene Expr Patterns *6*, 299-309.

S7. Aberle, H., Haghighi, A.P., Fetter, R.D., McCabe, B.D., Magalhaes, T.R., and Goodman, C.S. (2002). Wishful thinking encodes a BMP type II receptor that regulates synaptic growth in Drosophila. Neuron *33*, 545-558.

S8. Cole, S.H., Carney, G.E., McClung, C.A., Willard, S.S., Taylor, B.J., and Hirsh, J. (2005). Two functional but noncomplementing Drosophila tyrosine decarboxylase genes: distinct roles for neural tyramine and octopamine in female fertility. J Biol Chem *280*, 14948-14955.

S9. Taghert, P.H., Hewes, R.S., Park, J.H., O'Brien, M.A., Han, M., and Peck, M.E. (2001). Multiple amidated neuropeptides are required for normal circadian locomotor rhythms in Drosophila. J Neurosci *21*, 6673-6686.

S10. Campbell, G., Goring, H., Lin, T., Spana, E., Andersson, S., Doe, C.Q., and Tomlinson, A. (1994). RK2, a glial-specific homeodomain protein required for embryonic nerve cord condensation and viability in Drosophila. Development *120*, 2957-2966.

S11. Hummel, T., Krukkert, K., Roos, J., Davis, G., and Klambt, C. (2000). Drosophila Futsch/22C10 is a MAP1B-like protein required for dendritic and axonal development. Neuron *26*, 357-370.

S12. Picot, M., Cusumano, P., Klarsfeld, A., Ueda, R., and Rouyer, F. (2007). Light activates output from evening neurons and inhibits output from morning neurons in the Drosophila circadian clock. PLoS Biol *5*, e315.

S13. Crisp, S.J., Evers, J.F., and Bate, M. (2011). Endogenous patterns of activity are required for the maturation of a motor network. J Neurosci *31*, 10445-10450.

S14. Marley, R., Giachello, C.N., Scrutton, N.S., Baines, R.A., and Jones, A.R. (2014). Cryptochrome-dependent magnetic field effect on seizure response in Drosophila larvae. Sci Rep *4*, 5799.

S15. Emery, P., Stanewsky, R., Helfrich-Forster, C., Emery-Le, M., Hall, J.C., and Rosbash, M. (2000). Drosophila CRY is a deep brain circadian photoreceptor. Neuron *26*, 493-504.

S16. Klarsfeld, A., Malpel, S., Michard-Vanhee, C., Picot, M., Chelot, E., and Rouyer, F. (2004). Novel features of cryptochrome-mediated photoreception in the brain circadian clock of Drosophila. J Neurosci *24*, 1468-1477.
S17. Nagel, G., Brauner, M., Liewald, J.F., Adeishvili, N., Bamberg, E., and Gottschalk, A. (2005). Light activation of channelrhodopsin-2 in excitable cells of Caenorhabditis elegans triggers rapid behavioral responses. Curr Biol *15*, 2279-2284.
S18. Inada, K., Kohsaka, H., Takasu, E., Matsunaga, T., and Nose, A. (2011). Optical dissection of neural circuits responsible for Drosophila larval locomotion with halorhodopsin. PLoS ONE *6*, e29019.
S19. Arrenberg, A.B., Del Bene, F., and Baier, H. (2009). Optical control of zebrafish behavior with halorhodopsin. Proc Natl Acad Sci USA *106*, 17968-17973.
S20. Pownser, L. (1935). The effects of temperature on the duration of the developmental stages of Drosophila melanogaster. Physiol Zool *8*, 474-520.
S21. Marley, R., and Baines, R.A. (2011). Increased persistent Na^+ current contributes to seizure in the slamdance bang-sensitive Drosophila mutant. J Neurophysiol *106*, 18-29.
S22. De Deyn, P.P., D'Hooge, R., Marescau, B., and Pei, Y.Q. (1992). Chemical models of epilepsy with some reference to their applicability in the development of anticonvulsants. Epilepsy Res *12*, 87-110.
S23. Hamani, C., and Mello, L.E. (2002). Spontaneous recurrent seizures and neuropathology in the chronic phase of the pilocarpine and picrotoxin model epilepsy. Neurol Res *24*, 199-209.
S24. Mackenzie, L., Medvedev, A., Hiscock, J.J., Pope, K.J., and Willoughby, J.O. (2002). Picrotoxin-induced generalised convulsive seizure in rat: changes in regional distribution and frequency of the power of electroencephalogram rhythms. Clin Neurophysiol *113*, 586-596.
S25. Lee, J., and Wu, C.F. (2002). Electroconvulsive seizure behavior in Drosophila: Analysis of the physiological repertoire underlying a stereotyped action pattern in bang-sensitive mutants. J Neurosci *22*, 11065-11079.
S26. Stilwell, G.E., Saraswati, S., Littleton, J.T., and Chouinard, S.W. (2006). Development of a Drosophila seizure model for in vivo high-throughput drug screening. Eur J Neurosci *24*, 2211-2222.
S27. Wong, K., Stewart, A., Gilder, T., Wu, N., Frank, K., Gaikwad, S., Suciu, C., Dileo, J., Utterback, E., Chang, K., et al. (2010). Modeling seizure-related behavioral and endocrine phenotypes in adult zebrafish. Brain Res *1348*, 209-215.
S28. Lin, W.H., Gunay, C., Marley, R., Prinz, A.A., and Baines, R.A. (2012). Activity-dependent alternative splicing increases persistent sodium current and promotes seizure. J Neurosci *32*, 7267-7277.
S29. Stacey, S.M., Muraro, N.I., Peco, E., Labbe, A., Thomas, G.B., Baines, R.A., and van Meyel, D.J. (2010). Drosophila glial glutamate transporter Eaat1 is regulated by fringe-mediated notch signaling and is essential for larval locomotion. J Neurosci *30*, 14446-14457.
